# Establishing Thermodynamic Graphs of Nitrogenous Radical Cations Abstracting Hydrogen Atoms and Their Applications in Photoredox Reactions

**DOI:** 10.3390/molecules30030435

**Published:** 2025-01-21

**Authors:** Xia Zhao, Yi-Lin Hou, Jun Yang, Xin-Hua Wang, Chong-Shan Hu, Xiao-Qing Zhu, Guang-Bin Shen

**Affiliations:** 1 College of Medical Engineering, Jining Medical University, Jining 272000, China; 2 Department of Chemistry, Nankai University, Tianjin 300071, China

**Keywords:** thermodynamic graphs, hydrogen atom abstraction ability, nitrogenous radical cations, hydrogen atom transfer reactions

## Abstract

Nitrogenous compounds have been extensively utilized as hydrogen atom transfer (HAT) catalysts in photoredox reactions, with nitrogenous radical cations being the actual hydrogen atom abstractors. Building upon our previous work, 120 thermodynamic graphs of nitrogenous radical cations abstracting hydrogen atoms, which encompass seven vital thermodynamic parameters, are designed and established to elucidate their redox characteristics. Furthermore, the applications of thermodynamic graphs to select appropriate photocatalysts, assess the feasibility of the HAT process, and diagnose the possible activation mechanism were discussed, which would enable the utilities of nitrogenous compounds as HAT catalysts or nitrogenous radical cations as hydrogen atom abstractors in photoredox reactions.

## 1. Introduction

**Scheme 1:**
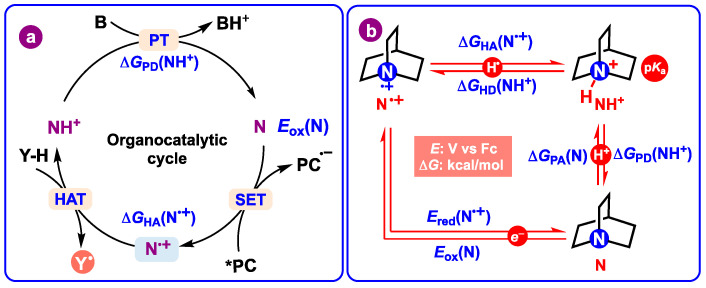
The representative mechanism for catalytic radical reactions involving N^•+^ functioning as HAT catalysts.

Nitrogenous compounds (N), such as quinuclidine (ABCO) [1,2,3,4,5,6], triethylamine (Et_3_N) [7,8,9], 1,4-diazabicyclo [2.2.2] octane (DABCO) [10,11,12,13], anilines [14,15]. and pyridines (Py) [16,17], have been widely utilized as hydrogen atom transfer (HAT) catalysts, particularly in photoredox reactions. It has been established that nitrogenous radical cations (N^•+^) are the actual hydrogen atom abstractors [1,2,3,4,5,6,7,8,9,10,11,12,13,14,15,16,17,18]. As illustrated in Scheme 1a, the formation of N^•+^ occurs via a single-electron transfer (SET) between the activated photocatalyst (*PC) and the nitrogenous compounds (N). Following this, N^•+^ abstracts a hydrogen atom from Y-H, yielding the key radical intermediate Y^•^ and transforming itself into NH^+^. The nitrogenous compound (N) is then regenerated through the neutralization reaction between NH^+^ and the base [19], completing the catalytic cycle.

Clearly, a single-electron oxidant is required to activate N into N^•+^. Consequently, for nitrogenous compounds (N), the oxidation potential of N is a crucial thermodynamic parameter for selecting an appropriate single-electron oxidant. For N^•+^, as an electron-deficient species, its hydrogen atom affinity and reduction potential are significant thermodynamic parameters that assess its ability to abstract a hydrogen atom and undergo single-electron oxidation. Lastly, for NH^+^, the Gibbs free energy associated with the proton donation by NH^+^ dictates the regeneration of the HAT catalysts [19].

In our previous work, the thermodynamic capabilities of various nitrogenous radical cations (N^•+^) abstracting hydrogen atoms were examined [20]. Building upon this, the thermodynamic graphs of nitrogenous radical cations abstracting hydrogen atoms (Scheme 1b), which incorporate seven thermodynamic parameters, are meticulously designed to properly and thoughtfully elucidate the redox characteristics of N^•+^, N, and NH^+^. Furthermore, the applications of thermodynamic graphs to select appropriate photocatalysts and diagnose or predict the possible activation mechanism were investigated.

## 2. Results and Discussion

**Scheme 2:**
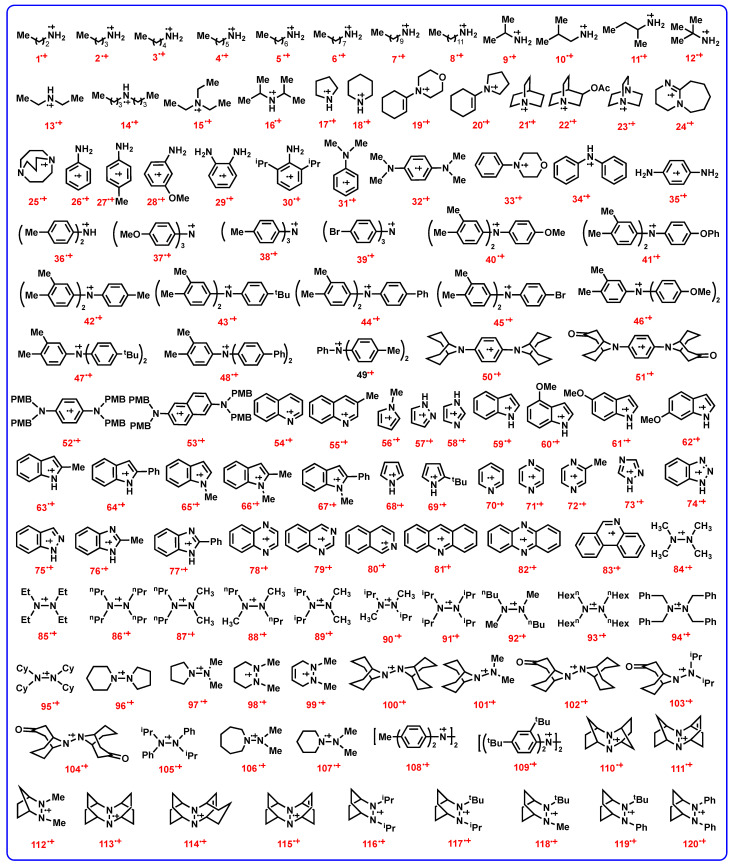
The chemical structures of 120 nitrogenous radical cations (N^•+^), consisting of 25 general amine radical cations (1^•+^~25^•+^), 28 aromatic amine radical cations (26^•+^~53^•+^), 30 aromatic N-heterocycle radical cations (54^•+^~83^•+^), and 37 tetra-substituted-hydrazine radical cations (84^•+^~120^•+^).

A total of 120 various nitrogenous radical cations (N^•+^), encompassing a broad range of compounds, such as general amine radical cations (1^•+^~25^•+^), aromatic amine radical cations (26^•+^~53^•+^), aromatic N-heterocycle radical cations (54^•+^~83^•+^), as well as tetra-substituted-hydrazine radical cations (84^•+^~120^•+^), are studied in this work. The chemical structures of these 120 nitrogenous radical cations (N^•+^) are depicted in Scheme 2.

### 2.1. Construction of Thermodynamic Graphs of N^•+^ Abstracting Hydrogen Atoms in Acetonitrile

**Scheme 3:**
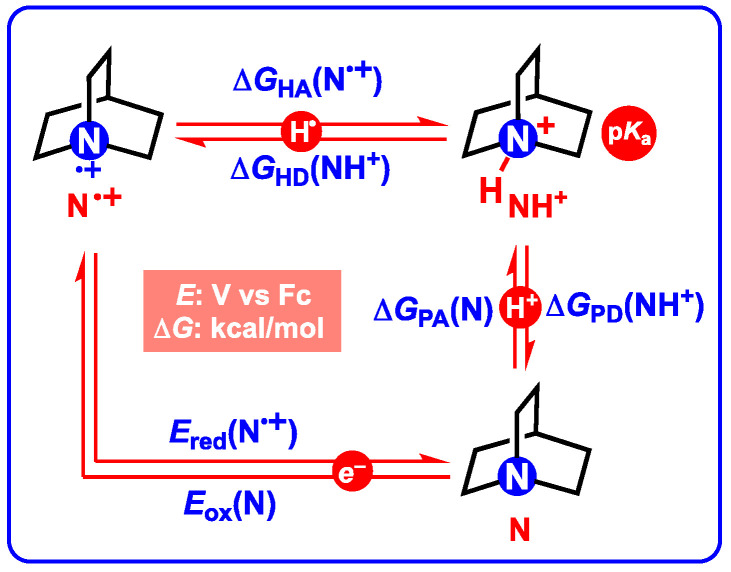
Thermodynamic graphs of N^•+^ abstracting hydrogen atoms in acetonitrile.

The constructed thermodynamic graphs of N^•+^ abstracting hydrogen atoms, which include seven key thermodynamic parameters, are presented in Scheme 3. From Scheme 3, Δ*G*_HA_(N^•+^) refers to the hydrogen atom affinity of N^•+^, while Δ*G*_HD_(NH^+^) denotes the Gibbs free energy of NH^+^ donating a hydrogen atom. *E*_red_(N^•+^) signifies the reduction potential of N^•+^, and *E*_ox_(N) represents the oxidation potential of N in acetonitrile (unit: V vs. Fc). The acidity of NH^+^ in acetonitrile is reflected by its p*K*_a_ value, denoted as p*K*_a_(NH^+^). Δ*G*_PD_(NH^+^) stands for the Gibbs free energy of NH^+^ donating a proton, and Δ*G*_PA_(N) corresponds to the Gibbs free energy change for N accepting a proton in acetonitrile.

The values for Δ*G*_HA_(N^•+^), p*K*_a_(NH^+^) and *E*_red_(N^•+^) have been previously reported in our work [20]. Herein, the Δ*G*_PA_(N) and Δ*G*_PD_(NH^+^) values are derived using the relationship with p*K*_a_(NH^+^), Δ*G*_PA_(N) = −Δ*G*_PD_(NH^+^) = −1.37p*K*_a_(NH^+^). *E*_ox_(N) values were determined by electrochemical technology in the literature, and the values are equivalent to *E*_red_(N^•+^) values, thus *E*_ox_(N) = *E*_red_(N^•+^). Given that the processes of N^•+^ abstracting a hydrogen atom and NH^+^ releasing a hydrogen atom are chemically opposite, the energy changes associated with these processes are exactly inverse. Consequently, Δ*G*_HD_(NH^+^) can be obtained from Δ*G*_HA_(N^•+^), Δ*G*_HD_(NH^+^) = −Δ*G*_HA_(N^•+^).

**Scheme 4:**
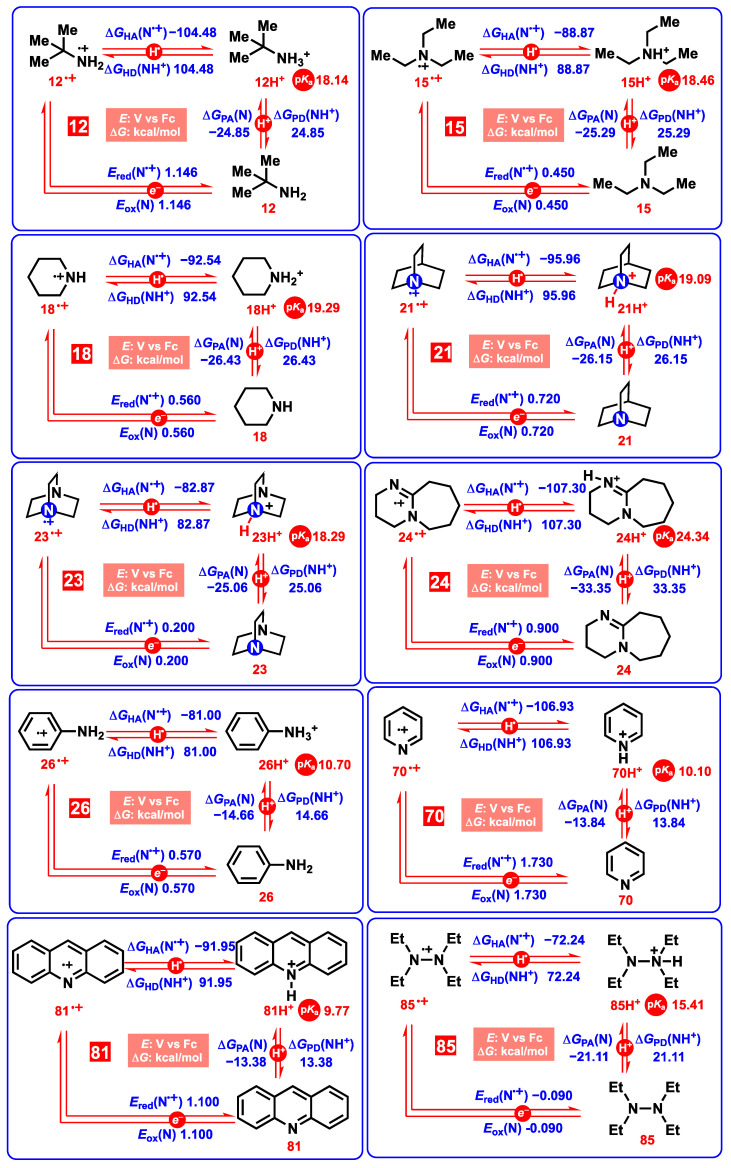
Representative thermodynamic graphs of N^•+^ abstracting H^•^ in acetonitrile.

As a result, 120 thermodynamic graphs [21,22,23] for a variety of various nitrogenous radical cations (N^•+^) are constructed and provided in the Supplementary Materials for chemists to query and use. Additionally, ten representative thermodynamic graphs for N^•+^ abstracting H^•^ (specifically 12^•+^, 15^•+^, 18^•+^, 21^•+^, 23^•+^, 24^•+^, 26^•+^, 70^•+^, 81^•+^ and 85^•+^) in acetonitrile are exhibited in Scheme 4. Undoubtedly, the thermodynamic graphs of N^•+^ abstracting hydrogen atoms reveal and exhibit the redox properties of N^•+^, N, and NH^+^, consisting of the single-electron oxidation and hydrogen atom abstraction capabilities of N^•+^, the single-electron reduction ability and basicity of N, as well as the acidity and antioxidant properties of NH^+^ in acetonitrile [24].

### 2.2. Correlations Among ΔG_HA_(N^•+^), E_ox_(N) and ΔG_PA_(N)

**Figure 1:**
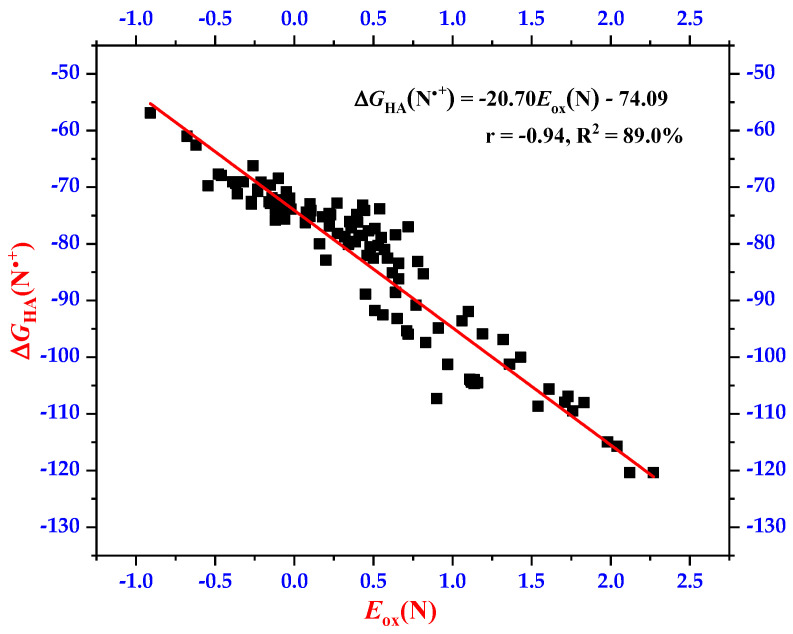
Relationship between Δ*G*_HA_(N^•+^) and *E*_ox_(N) of 120 nitrogenous radical cations (N^•+^), along with the reduction potentials of the frequently used oxidative single-electron photocatalysts.

As previously discussed, the nitrogenous compound (N) must first be activated by single-electron oxidants to form nitrogenous radical cations (N^•+^), which are the actual hydrogen atom abstractor in chemical reactions [1,2,3,4,5,6,7,8,9,10,11,12,13,14,15,16,17,18,19]. Accordingly, the oxidation potential of nitrogenous compounds (N) is a crucial thermodynamic parameter that determines the single-electron activated difficulty level of N. Figure 1 illustrates the relationship between Δ*G*_HA_(N^•+^) and *E*_ox_(N) for 120 nitrogenous compounds (N) in acetonitrile. Notably, a strong linear correlation is observed between Δ*G*_HA_(N^•+^) and *E*_ox_(N) for these 120 nitrogenous compounds (N). The higher the *E*_ox_(N) is, the more negative the hydrogen atom affinity of N^•+^ is, and the stronger the hydrogen atom abstraction ability of N^•+^ is. The fitted linear equation is Δ*G*_HA_(N^•+^) = −20.70*E*_ox_(N) − 74.09, with a correlation coefficient (r) of −0.94 and an R-squared value of 89.0%. This equation suggests that the hydrogen atom affinity of N^•+^ can be approximately predicted if the oxidation potential of the nitrogenous compound (N) is known. This unexpected linear relationship provides a rudimentary method for selecting a suitable nitrogenous compound as a hydrogen atom transfer (HAT) catalyst or choosing a nitrogenous radical cation (N^•+^) as a hydrogen atom abstractor in Y-H functionalization reactions.

**Figure 2:**
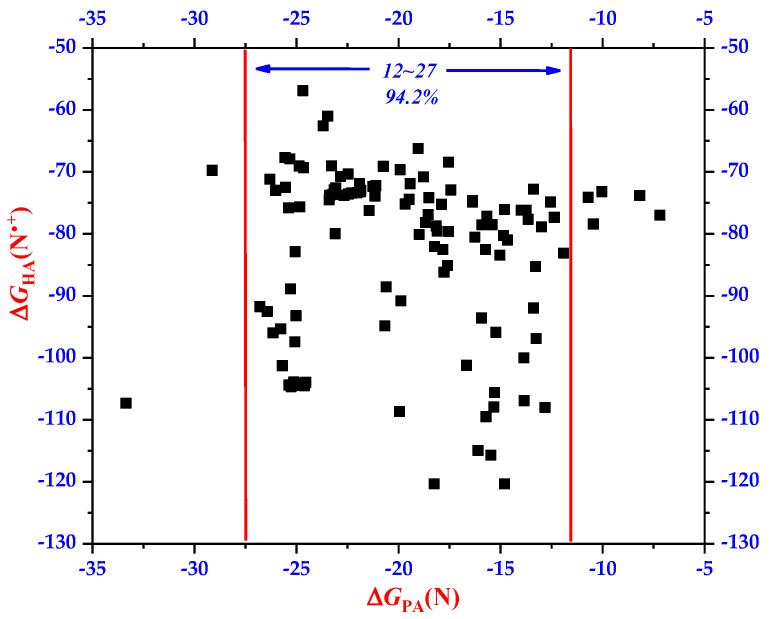
Relationship between Δ*G*_HA_(N^•+^) and Δ*G*_PA_(N) of 120 nitrogenous radical cations (N^•+^).

**Figure 3:**
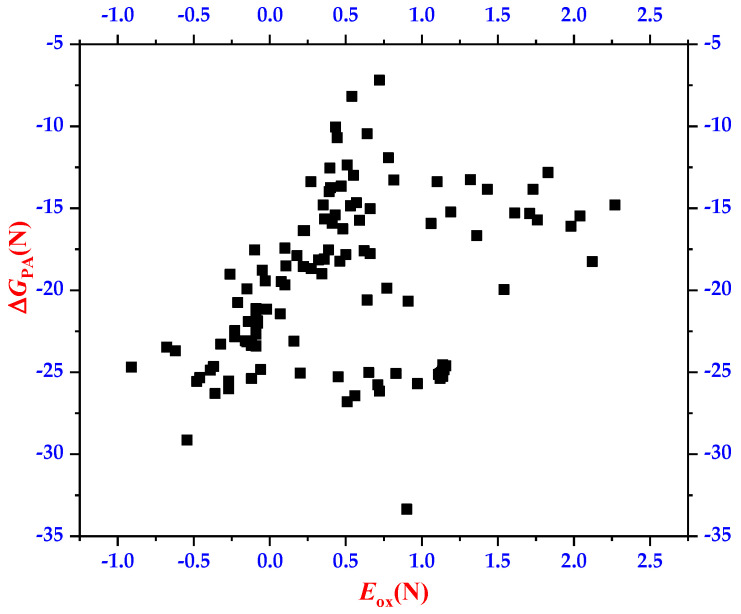
Relationship between Δ*G*_PA_(N) and *E*_ox_(N) of 120 nitrogenous radical cations (N^•+^).

Moreover, the relationship between Δ*G*_HA_(N^•+^) and Δ*G*_PA_(N) of 120 nitrogenous radical cations (N^•+^) (Figure 2), and the relationship between Δ*G*_PA_(N) and *E*_ox_(N) of 120 nitrogenous compounds (N^•+^) are also explored (Figure 3). Interestingly, no linear correlation was identified in these relationships. The absence of a linear relationship between Δ*G*_HA_(N^•+^) and Δ*G*_PA_(N) (Figure 2) underscores that the oxidation potential of a nitrogenous compound [*E*_ox_(N)], rather than proton affinity [Δ*G*_PA_(N)], is the pivotal factor influencing the hydrogen atom affinity of the corresponding nitrogenous radical cation (N^•+^). Because of the absence of a linear relationship between Δ*G*_PA_(N) and *E*_ox_(N) (Figure 3), it is believed that the proton affinity of a N is determined by its basicity instead of its oxidation potential.

### 2.3. Application of Thermodynamic Graphs to Easily Compare Thermodynamic Properties of N^•+^, N and NH^+^

**Scheme 5:**
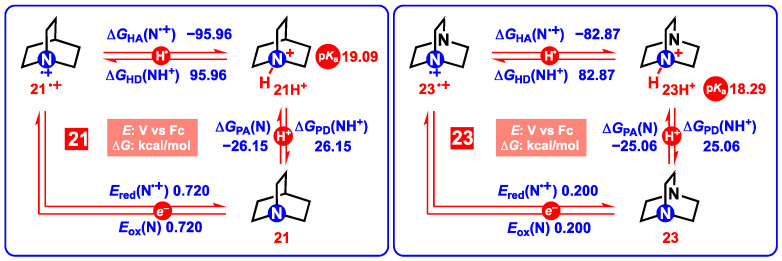
Comparison of thermodynamic properties of N^•+^, N and NH^+^ between 21^•+^ and 23^•+^.

The thermodynamic graphs of N^•+^ accepting hydrogen atoms reveal the redox properties of N^•+^, N and NH^+^ in acetonitrile, which are crucial for understanding and applying these species in chemical reactions. For instance, as shown in Scheme 5, the hydrogen atom affinity of 21^•+^ (−95.96 kcal/mol) is 13.09 kcal/mol larger than 23^•+^ (−82.87 kcal/mol), which means 21^•+^ [1,2,3,4,5,6] is a thermodynamically more effective hydrogen atom abstractor than 23^•+^ [10,11,12,13]. In terms of NH^+^, the hydrogen-atom-donating ability of 21H^+^ (Δ*G*_HD_ = 95.96 kcal/mol) is 13.09 kcal/mol less than 23H^+^ (Δ*G*_HD_ = 82.87 kcal/mol), suggesting that 21H^+^ is a thermodynamically less potent antioxidant than 23H^+^ as a hydrogen atom donor.

Although both 21^•+^ (0.720 V vs. Fc) and 23^•+^ (0.200 V vs. Fc) are medium–strong single-electron oxidants, the single-electron oxidation ability of 21^•+^ is 0.520 V (vs. Fc, equal to 12.0 kcal/mol) stronger than that of 23^•+^, indicating that 21^•+^ is a thermodynamically better single-electron oxidant than 21^•+^ in acetonitrile. Instead, both 21 and 23 belong to medium–strong single-electron reductants, and the single-electron reduction ability of 21 (0.720 V vs. Fc) is 0.520 V (vs Fc, equal to 12.0 kcal/mol) weaker than 23 (0.200 V vs. Fc).

For NH^+^, the acidity of 21H^+^ (p*K*_a_ = 19.09) is slightly weaker than 23H^+^ (p*K*_a_ = 18.29) in acetonitrile, and they both belong to medium–strong organic acids, implying that a moderate–strong base with a p*K*_a_ larger than 19.5 is required to regenerate N as a HAT catalyst [24]. In contrast, the basicity of 21 (Δ*G*_PA_ = −26.15 kcal/mol) is slightly stronger than 23 (Δ*G*_PA_ = −25.06 kcal/mol) by 1.09 kcal/mol in acetonitrile, and they both belong to a medium–strong organic base.

In summary, the thermodynamic graphs, comprising seven critical thermodynamic parameters, offer a precious tool for easily comparing the thermodynamic properties of N^•+^, N and NH^+^. This enables the strategic application of nitrogenous compounds (N) as HAT catalysts or nitrogenous radical cations (N^•+^) as hydrogen atom abstractors in Y-H functionalization reactions.

### 2.4. Application of Thermodynamic Graphs to Select Suitable Photocatalysts to Activate N into N^•+^ in Photoredox Reactions

As detailed in 120 thermodynamic graphs provided in the Supporting Materials, the oxidation potential values [*E*_ox_(N)] of 120 nitrogenous compounds (N) cover a very wide range of 3.18 V, spanning from -0.91 V (**113**) to 2.27 V vs. Fc (73) in acetonitrile. The oxidation potentials of nitrogenous compounds are closely related to their chemical structures. Generally, the oxidation potentials of nitrogenous compounds increase according the following order of aromatic N-heterocycle radical cations (54^•+^~83^•+^) ≈ general amine radical cations (1^•+^~25^•+^) > aromatic amine radical cations (26^•+^~53^•+^) > tetra-substituted-hydrazine radical cations (84^•+^~120^•+^). The oxidation potentials of nitrogenous compounds [*E*_ox_(N)] provide us with a precise guidance for selecting appropriate single-electron oxidants to activate nitrogenous compounds (N, HAT catalysts) into N^•+^ (the real hydrogen atom abstractors).

**Scheme 6:**
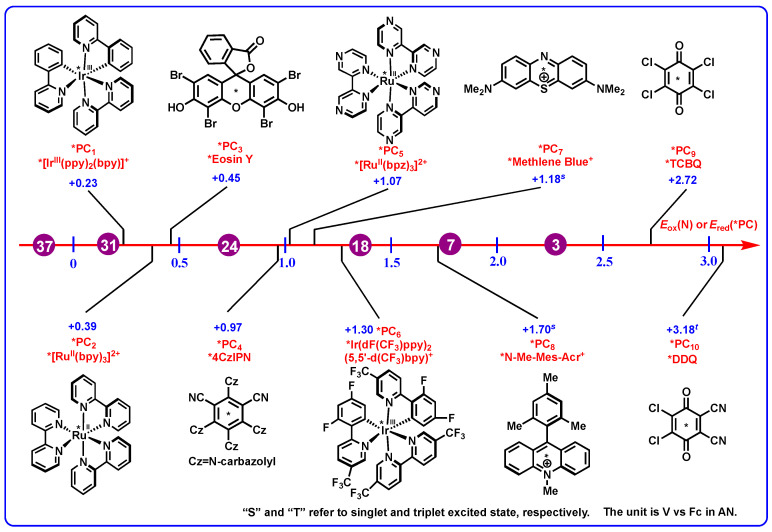
Chemical structures and oxidation potentials of frequently used oxidative single-electron photocatalysts, along with the oxidation potentials distribution (the white data in purple circle).

Examining previous works, photocatalysts have always been used to construct photocatalyst/N pairs [1,2,3,4,5,6,7,8,9,10,11,12,13,14,15,16,17,18]. After SET between photocatalysts and N, nitrogenous compounds (N) were converted into nitrogenous radical cations (N^•+^) to initiate the following HAT and radical reactions. With the recent surge in the development of photochemical reactions [25,26,27,28,29,30], numerous oxidative single-electron photocatalysts have been developed to induce or initiate radical reactions [31,32,33]. In this context, the reduction potentials of ten frequently used photocatalysts, including four transition metal photocatalysts [*PC_1_, *PC_2_, *PC_5_ and *PC_6_] and six metal-free organic photocatalysts [*PC_3_, *PC_4_ and *PC_7_-*PC_10_] [31,32,33], were compiled and presented in Scheme 6. As evident in Scheme 6, the ten photocatalysts exhibit strong single-electron oxidation capabilities, and the reduction potentials of 10 photocatalysts cover a wide range of 2.95 V, spanning from +0.23 V (*PC_1_) to +3.18 V vs. Fc (*PC_10_). For a specific nitrogenous compounds (N), a few suitable photocatalysts can be conveniently and efficiently selected from a thermodynamic perspective, aided by the thermodynamic graphs and Scheme 6, and the potentials should meet the following conditions of *E*_red_(*PC) > *E*_ox_(N).

To guide the selection of photocatalysts more effectively, the oxidation potentials distribution of 120 nitrogenous compounds (N) are also depicted in Scheme 6. It is found that (1) the oxidation potentials of 37 nitrogenous compounds are more negative than 0 V (vs. Fc), meaning that they are thermodynamically very strong single-electron donors and very easy to be activated. (2) The oxidation potentials of 55 nitrogenous compounds range between 0 V and 1.0 V (vs. Fc), indicating that they are thermodynamically medium–strong single-electron donors and easily activated. (3) The oxidation potentials of 25 nitrogenous compounds are greater than 1.0 V and more negative than 2.0 V (vs. Fc), suggesting that they belong to thermodynamically weak single-electron donors and are difficult to activate. (4) Only three nitrogenous compounds have oxidation potentials greater than 2.0 V (vs. Fc), which imply that they are thermodynamically very weak single-electron donors and very difficult to be activated.

Based on the oxidation potentials distribution for the 120 nitrogenous compounds (N) and the reduction potentials of ten frequently used photocatalysts, several valuable conclusions can be drawn, as follows: (1) Since the oxidation potentials of 120 nitrogenous compounds (N) are all below 2.5 V (vs. Fc), *PC_9_ (2.72 V vs. Fc) and *PC_10_ (3.18*^T^* V vs. Fc) are capable of activating them into N^•+^. (2) Over 91% (110 out of 120) of the nitrogenous compounds (N) have oxidation potentials less than 1.5 V (vs. Fc), and *PC_8_ (1.70*^S^* V vs. Fc), *PC_9_ (2.72 V vs. Fc) and *PC_10_ (3.18*^T^* V vs. Fc) could efficiently activate them into N^•+^. (3) Classical photocatalysts such as *PC_1_ (0.23 V vs. Fc), *PC_2_ (0.39 V vs. Fc), and *PC_5_ (0.45 V vs. Fc) can only activate a portion (no more than 58 out of 120) of the nitrogenous compounds (N) into N^•+^. (4) The broad potential distributions of nitrogenous compounds (N) and frequently used photocatalysts further verified the importance of thermodynamic graphs and Scheme 6. It is believed that a combination of thermodynamic graphs and Scheme 6 serves as a significant instructional tool to assist synthetic chemists in selecting efficient photocatalysts to activate nitrogenous compounds (N, HAT catalysts) into N^•+^ species (the real hydrogen atom abstractors) by constructing *PC/N pair [1,2,3,4,5,6,7,8,9,10,11,12,13,14,15,16,17,18]. Moreover, when a photocatalyst is fixed, the combination of thermodynamic graphs and Scheme 6 can also guide chemists in choosing efficient nitrogenous compounds as HAT catalysts, whose N^•+^ species possess strong hydrogen atom abstracting abilities. That is, HAT catalysts and the single-electron photoexcited initiators can be efficiently and precisely designed through constructing suitable *PC/N pairs under various chemical reactions, with the help of thermodynamic graphs and Scheme 6.

### 2.5. Application of Thermodynamic Graphs to Assess the Feasibility of the HAT Process and Diagnose the Possible Activation Mechanism

Since the thermodynamic graphs of nitrogenous radical cations abstracting hydrogen atoms unveil the thermodynamic redox properties of N^•+^, N, and NH^+^, they can be utilized to assess the feasibility of the HAT process and to diagnose the possible activation mechanism from a thermodynamic view. According to thermodynamic graphs, N^•+^ simultaneously possess single-electron and hydrogen atom abstracting abilities, with the corresponding thermodynamic forces determined by the *E*_red_(N^•+^) and Δ*G*_HA_(N^•+^) values, respectively. Herein, the activation processes between 21^•+^ and toluene (TH), as well as 21^•+^ and 9*H*-xanthene (OH), are taken as examples to demonstrate the promising applications of thermodynamic graphs.

**Scheme 7:**
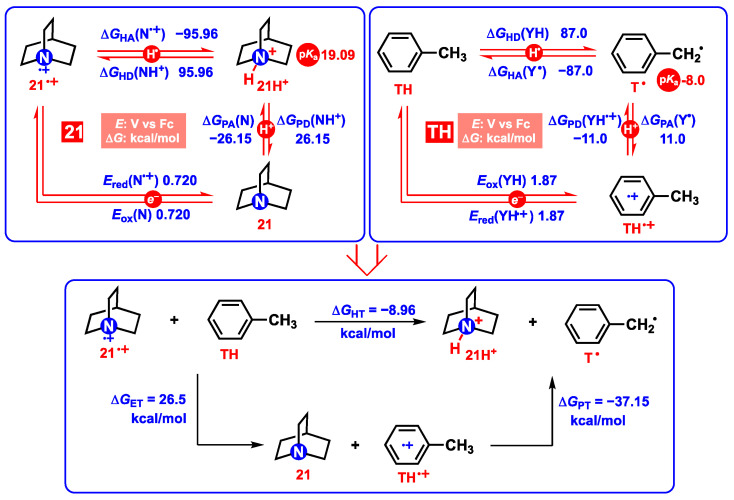
Thermodynamic analysis of 21^•+^ abstracting a hydrogen atom from toluene (TH).

For the activation process involving 21^•+^ and toluene (TH), the thermodynamic graphs of 21^•+^ abstracting a hydrogen atom and toluene (TH) donating a hydrogen atom [34] are shown in Scheme 7 to facilitate a thorough thermodynamic analysis. The activation process may proceed a direct HAT or successive electron and proton transfer mechanism. Deciphering which one is the actual activation mechanism needs further thermodynamic discussion. From Scheme 7, it is observed that the Gibbs free energy for the HAT process [Δ*G*_HT_(21^•+^/TH)] is −8.96 kcal/mol, meaning the HAT process between 21^•+^ and toluene (TH) is thermodynamically feasible. Given that 21^•+^ is also a medium–strong single-electron acceptor (*E*_red_ = 0.720 V vs. Fc), if the single-electron transfer (SET) between 21^•+^ and TH occurs, the associated thermodynamic driving force [Δ*G*_ET_(21^•+^/TH)] would be 26.5 kcal/mol. Although the subsequent proton transfer from TH^•+^ to 21 is thermodynamically highly favorable with Δ*G*_PT_(21/TH^•+^) being −37.15 kcal/mol, the initiating SET is impossible. Obviously, 26.5 kcal/mol represents a very high energy barrier, which is 35.46 kcal/mol higher than that of the direct HAT process [Δ*G*_HT_(21^•+^/TH) = −8.96 kcal/mol]. Taking all three thermodynamic driving forces into consideration, the activation process between 21^•+^ and toluene (TH) is thermodynamically feasible [Δ*G*_HT_(21^•+^/TH) = −8.96 kcal/mol], and it is likely to proceed via a direct HAT mechanism.

**Scheme 8:**
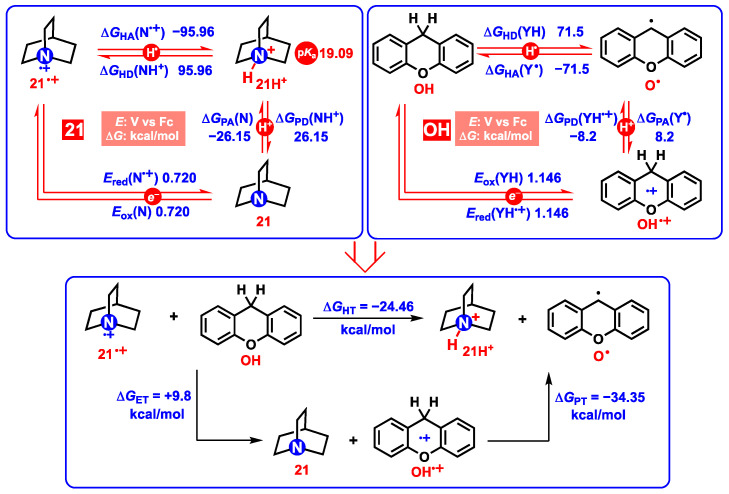
Thermodynamic analysis of 21^•+^ abstracting a hydrogen atom from 9*H*-xanthene (OH).

Xanthenes are a type of biologically active molecules [35] and fluorescent materials [36,37]. Recently, many groups focused on the method development to synthetize diverse derivatives [38], especially their functionalization for the 9-position of xanthenes [39,40,41,42,43]. It has been reported that the radical activation modes primarily include two pathways: direct HAT [39,40,41,42] and successive electron and proton transfer [43]. For the activation process of 21^•+^ and 9*H*-xanthene (OH), the thermodynamic graphs depicting 21^•+^ abstracting a hydrogen atom and 9*H*-xanthene (OH) donating a hydrogen atom [34] are presented in Scheme 8 for a more detailed thermodynamic analysis. From Scheme 8, the Gibbs free energy of HAT process [Δ*G*_HT_(21^•+^/OH)] is −24.46 kcal/mol, meaning the HAT process between them is thermodynamically feasible. Additionally, if the SET takes place between 21^•+^ and xanthene (OH), the corresponding thermodynamic driving force [Δ*G*_ET_(21^•+^/OH)] is 9.8 kcal/mol, which is a very low barrier to overcome. Moreover, the subsequent proton transfer from OH^•+^ to 21 is thermodynamically highly favorable, with Δ*G*_PT_(21/OH^•+^) being −34.35 kcal/mol. Considering these three thermodynamic driving forces, the activation process between 21^•+^ and 9*H*-xanthene (OH) is thermodynamically feasible [Δ*G*_HT_(21^•+^/TH) = −8.96 kcal/mol], and it may experience a direct HAT or successive electron and proton transfer mechanism, or both processes, from a thermodynamic view [39,40,41,42,43]. Further laboratory verification will be required to determine the actual mechanism.

It can be seen that the thermodynamic analyses for the activation processes between 21^•+^ and toluene (TH), 21^•+^ and 9*H*-xanthene (OH) provide us with the other possibility that the activation process may experience a successive electron and proton transfer mechanism beyond direct HAT route. The published work also verified this opinion [39,40,41,42,43]. These two examples also exhibit the valuable application of thermodynamic graphs to assess the feasibility of the HAT process and diagnose the possible activation mechanism.

It should be noted that in the HAT process involving the nitrogenous radical cation (N^•+^) and the substrate Y-H, sometimes an EDA (electron donor–acceptor) complex of N^•+^ and Y-H may form when the substrate possesses an appropriate electron-rich structure. The interaction within the EDA complex may alter the hydrogen atom affinity of N^•+^ compared to its state in a free solution, as well as the Gibbs free energy of Y-H donating a hydrogen atom. However, the overall free energy change for the HAT process can be assessed by considering the thermodynamic values of hydrogen atom affinity of N^•+^ and the Gibbs free energy of Y-H donating a hydrogen atom.

## 3. Experimental Section

The thermodynamic parameters are depicted in thermodynamic graphs; the Δ*G*_HA_(N^•+^), p*K*_a_(NH^+^) and *E*_red_(N^•+^) values are available from our previous work. The Δ*G*_PA_(N) and Δ*G*_PD_(NH^+^) values could be derived from p*K*_a_(NH^+^), Δ*G*_PA_(N) = −Δ*G*_PD_(NH^+^) = −1.37p*K*_a_(NH^+^). *T*he *E*_ox_(N) values are equal to *E*_red_(N^•+^) values, *E*_ox_(N) = *E*_red_(N^•+^). Δ*G*_HD_(NH^+^) can be obtained from Δ*G*_HA_(N^•+^), Δ*G*_HD_(NH^+^) = −Δ*G*_HA_(N^•+^).

## 4. Conclusions

In summary, building upon our previous work, 120 thermodynamic graphs of nitrogenous radical cations abstracting hydrogen atoms, which encompass seven vital thermodynamic parameters, have been meticulously designed and established to exhibit the redox properties of N^•+^, N, and NH^+^. A strong linear correlation was observed between Δ*G*_HA_(N^•+^) and *E*_ox_(N) for these 120 nitrogenous compounds (N), suggesting that the hydrogen atom affinity of N^•+^ can be approximately predicted if the oxidation potential of a N is known. Furthermore, the applicability of thermodynamic graphs to select appropriate photocatalysts, assess the feasibility of the HAT process, and diagnose the possible activation mechanism was investigated. It is believed that the research results can enable the strategic application of nitrogenous compounds as HAT catalysts, or nitrogenous radical cations as hydrogen atom abstractors, in photo-promoted Y-H functionalization reactions.

## Data Availability

The underlying data from this study are available in the published article.

## Supplementary Materials

The following supporting materials can be downloaded at: https://www.mdpi.com/article/10.3390/molecules30030435/s1. Table S1. The *E*_red_(N^•+^) and Δ*G*_HA_(N^•+^) values of N^•+^, *E*_ox_(N) and Δ*G*_PA_(N) values of N, as well as the p*K*_a_(NH^+^), Δ*G*_PD_(NH^+^) and Δ*G*_HD_(NH^+^) values of NH^+^ in acetonitrile (the unit of *E* is V vs. Fc, and the unit of Δ*G* is kcal/mol). Thermodynamic graphs of 120 N^•+^.
