# Supplementary material for: Establishing Thermodynamic Graphs of Nitrogenous Radical Cations Abstracting Hydrogen Atoms and Their Applications in Photoredox Reactions
Source: Molecules. 2025 Jan 21;30(3):435. doi: 10.3390/molecules30030435 (PMC11819841; doi:10.3390/molecules30030435)
Supplement: Supplementary file 1 [file molecules-30-00435-s001.zip › molecules-3326339-supplementary.pdf]

Establishing Thermodynamic Graphs of Nitrogenous Radical Cations Abstracting Hydrogen Atoms and Their Applications in Photoredox Reactions

Xia Zhao [1], Yi-Lin Hou [1], Jun Yang [1], Xin-Hua Wang [1], Chong-Shan Hu [1], Xiao-Qing Zhu [2,*] and Guang-Bin Shen [1,*]

[1] *College of Medical Engineering, Jining Medical University, Jining, Shandong, 272000, P. R. China.*

[2] *Department of Chemistry, Nankai University, Tianjin 300071, China*

** Correspondence: xqzhu@nankai.edu.cn (X.-Q.Z.); gbshen@mail.jnmc.edu.cn (G.-B.S.)*

Contents

Table S1...*S2*

Thermodynamic graphs of 120 $N^{\bullet+}$..*S5*

Table S1. $E_{red}(N^{\bullet+})$ and $\Delta G_{HA}(N^{\bullet+})$ values of $N^{\bullet+}$, $E_{ox}(N)$ and $\Delta G_{PA}(N)$ values of N, as well as the $pK_a(NH^+)$, $\Delta G_{PD}(NH^+)$ and $\Delta G_{HD}(NH^+)$ values of NH^+ in acetonitrile (the unit of E is V vs Fc, and the unit of ΔG is kcal/mol)

NO.	$N^{\bullet+}$	$E_{red}(N^{\bullet+})^a$ / $E_{ox}(N)^b$	$pK_a(NH^+)^a$	$\Delta G_{PA}(N)^c$ / $-\Delta G_{PD}(NH^+)^c$	$\Delta G_{HA}(N^{\bullet+})^a$ / $-\Delta G_{HD}(NH^+)^d$
1	$1^{\bullet+}$	1.137	18.43	-25.25	-104.67
2	$2^{\bullet+}$	1.129	18.26	-25.02	-104.25
3	$3^{\bullet+}$	1.120	18.30	-25.07	-104.10
4	$4^{\bullet+}$	1.109	18.34	-25.13	-103.90
5	$5^{\bullet+}$	1.116	18.35	-25.14	-104.07
6	$6^{\bullet+}$	1.121	18.35	-25.14	-104.19
7	$7^{\bullet+}$	1.123	18.35	-25.14	-104.24
8	$8^{\bullet+}$	1.120	18.52	-25.37	-104.40
9	$9^{\bullet+}$	1.158	17.96	-24.61	-104.51
10	$10^{\bullet+}$	1.135	18.20	-24.93	-104.31
11	$11^{\bullet+}$	1.137	17.92	-24.55	-103.97
12	$12^{\bullet+}$	1.146	18.14	-24.85	-104.48
13	$13^{\bullet+}$	0.970	18.75	-25.69	-101.26
14	$14^{\bullet+}$	0.830	18.31	-25.08	-97.42
15	$15^{\bullet+}$	0.450	18.46	-25.29	-88.87
16	$16^{\bullet+}$	0.710	18.81	-25.77	-95.34
17	$17^{\bullet+}$	0.510	19.56	-26.80	-91.76
18	$18^{\bullet+}$	0.560	19.29	-26.43	-92.54
19	$19^{\bullet+}$	0.160	16.86	-23.10	-79.99
20	$20^{\bullet+}$	-0.060	17.40	-24.84	-75.65
21	$21^{\bullet+}$	0.720	19.09	-26.15	-95.96
22	$22^{\bullet+}$	0.650	18.26	-25.02	-93.20
23	$23^{\bullet+}$	0.200	18.29	-25.06	-82.87
24	$24^{\bullet+}$	0.900	24.34	-33.35	-107.30
25	$25^{\bullet+}$	-0.545	21.26	-29.13	-69.76
26	$26^{\bullet+}$	0.570	10.70	-14.66	-81.00
27	$27^{\bullet+}$	0.430	11.25	-15.41	-78.53
28	$28^{\bullet+}$	0.480	11.86	-16.25	-80.52
29	$29^{\bullet+}$	0.100	12.72	-17.43	-72.93
30	$30^{\bullet+}$	0.460	13.31	-18.23	-82.04
31	$31^{\bullet+}$	0.360	11.43	-15.66	-77.16
32	$32^{\bullet+}$	-0.260	13.89	-19.03	-66.23
33	$33^{\bullet+}$	0.500	13.01	-17.82	-82.55
34	$34^{\bullet+}$	0.540	5.97	-8.18	-73.83
35	$35^{\bullet+}$	0.106	13.51	-18.51	-74.15
36	$36^{\bullet+}$	0.400	10.04	-13.75	-76.18
37	$37^{\bullet+}$	0.180	13.06	-17.89	-75.24
38	$38^{\bullet+}$	0.395	9.16	-12.55	-74.86
39	$39^{\bullet+}$	0.720	5.25	-7.19	-77.00
40	$40^{\bullet+}$	0.273	13.63	-18.67	-78.17

NO.	$N^{•+}$	$E_{red}(N^{•+})^a$ / $E_{ox}(N)^b$	$pK_a(NH^+)^a$	$\Delta G_{PA}(N)^c$ / $-\Delta G_{PD}(NH^+)^c$	$\Delta G_{HA}(N^{•+})^a$ / $-\Delta G_{HD}(NH^+)^d$
41	$41^{•+}$	0.343	13.86	-18.99	-80.10
42	$42^{•+}$	0.350	10.80	-14.80	-76.07
43	$43^{•+}$	0.359	13.21	-18.10	-79.58
44	$44^{•+}$	0.390	10.21	-13.99	-76.18
45	$45^{•+}$	0.470	9.96	-13.65	-77.68
46	$46^{•+}$	0.222	13.54	-18.55	-76.87
47	$47^{•+}$	0.386	12.80	-17.54	-79.64
48	$48^{•+}$	0.443	7.81	-10.70	-74.11
49	$49^{•+}$	0.432	7.33	-10.04	-73.20
50	$50^{•+}$	-0.360	19.20	-26.30	-71.20
51	$51^{•+}$	-0.090	16.39	-22.45	-73.58
52	$52^{•+}$	-0.030	14.18	-19.43	-71.93
53	$53^{•+}$	0.076	14.22	-19.48	-74.43
54	$54^{•+}$	1.760	11.47	-15.71	-109.50
55	$55^{•+}$	1.190	11.13	-15.23	-95.89
56	$56^{•+}$	0.660	12.97	-17.77	-86.19
57	$57^{•+}$	1.830	9.21	-12.82	-108.02
58	$58^{•+}$	0.770	14.51	-19.88	-90.83
59	$59^{•+}$	0.780	8.70	-11.92	-83.11
60	$60^{•+}$	0.590	11.48	-15.73	-82.53
61	$61^{•+}$	0.660	10.97	-15.03	-83.45
62	$62^{•+}$	0.530	10.84	-14.85	-80.27
63	$63^{•+}$	0.510	9.02	-12.36	-77.32
64	$64^{•+}$	0.640	7.63	-10.45	-78.41
65	$65^{•+}$	0.816	9.69	-13.28	-85.29
66	$66^{•+}$	0.410	11.61	-15.91	-78.56
67	$67^{•+}$	0.550	9.48	-12.99	-78.87
68	$68^{•+}$	0.620	12.84	-17.59	-85.09
69	$69^{•+}$	0.640	15.04	-20.60	-88.56
70	$70^{•+}$	1.730	10.10^e	-13.84	-106.93
71	$71^{•+}$	2.040	11.30	-15.48	-115.72
72	$72^{•+}$	1.980	11.75	-16.10	-114.96
73	$73^{•+}$	2.270	10.80	-14.80	-120.34
74	$74^{•+}$	1.610	11.16	-15.29	-105.62
75	$75^{•+}$	1.060	11.63	-15.93	-93.58
76	$76^{•+}$	0.910	15.09	-20.67	-94.86
77	$77^{•+}$	1.540	14.56	-19.95	-108.66
78	$78^{•+}$	1.710	11.18	-15.32	-107.95
79	$79^{•+}$	2.120	13.32	-18.25	-120.34
80	$80^{•+}$	1.360	12.17	-16.67	-101.23
81	$81^{•+}$	1.100	9.77	-13.38	-91.95
82	$82^{•+}$	1.430	10.11	-13.85	-100.03
83	$83^{•+}$	1.320	9.67	-13.25	-96.89

NO.	$N^{\bullet+}$	$E_{red}(N^{\bullet+})^a$ / $E_{ox}(N)^b$	$pK_a(NH^+)^a$	$\Delta G_{PA}(N)^c$ / $-\Delta G_{PD}(NH^+)^c$	$\Delta G_{HA}(N^{\bullet+})^a$ / $-\Delta G_{HD}(NH^+)^d$
84	$84^{\bullet+}$	-0.100	12.80	-17.54	-68.43
85	$85^{\bullet+}$	-0.090	15.41	-21.11	-72.24
86	$86^{\bullet+}$	-0.090	16.27	-22.29	-73.41
87	$87^{\bullet+}$	-0.080	16.07	-22.02	-73.37
88	$88^{\bullet+}$	-0.080	15.96	-21.87	-73.22
89	$89^{\bullet+}$	-0.090	15.95	-21.85	-72.98
90	$90^{\bullet+}$	-0.140	15.99	-21.91	-71.88
91	$91^{\bullet+}$	-0.120	16.95	-23.22	-73.65
92	$92^{\bullet+}$	-0.090	16.55	-22.67	-73.80
93	$93^{\bullet+}$	-0.090	15.51	-21.25	-72.37
94	$94^{\bullet+}$	0.220	11.95	-16.37	-74.64
95	$95^{\bullet+}$	-0.120	17.06	-23.37	-73.80
96	$96^{\bullet+}$	-0.230	16.68	-22.85	-70.75
97	$97^{\bullet+}$	-0.210	15.14	-20.74	-69.10
98	$98^{\bullet+}$	-0.150	14.54	-19.92	-69.66
99	$99^{\bullet+}$	-0.050	13.70	-18.77	-70.82
100	$100^{\bullet+}$	-0.390	18.15	-24.87	-69.07
101	$101^{\bullet+}$	-0.270	18.99	-26.02	-72.99
102	$102^{\bullet+}$	-0.160	16.85	-23.08	-72.59
103	$103^{\bullet+}$	-0.090	17.08	-23.40	-74.52
104	$104^{\bullet+}$	0.070	15.65	-21.44	-76.25
105	$105^{\bullet+}$	0.320	13.25	-18.15	-78.73
106	$106^{\bullet+}$	-0.150	16.90	-23.15	-72.89
107	$107^{\bullet+}$	-0.020	15.44	-21.15	-73.89
108	$108^{\bullet+}$	0.270	9.77	-13.38	-72.81
109	$109^{\bullet+}$	0.230	11.95	-16.37	-74.87
110	$110^{\bullet+}$	-0.370	18.00	-24.66	-69.33
111	$111^{\bullet+}$	-0.322	16.99	-23.28	-69.05
112	$112^{\bullet+}$	-0.230	16.40	-22.47	-70.36
113	$113^{\bullet+}$	-0.910	18.02	-24.69	-56.90
114	$114^{\bullet+}$	-0.678	17.13	-23.47	-61.03
115	$115^{\bullet+}$	-0.621	17.29	-23.69	-62.57
116	$116^{\bullet+}$	-0.460	18.49	-25.33	-67.92
117	$117^{\bullet+}$	-0.480	18.66	-25.56	-67.69
118	$118^{\bullet+}$	-0.270	18.64	-25.54	-72.51
119	$119^{\bullet+}$	-0.120	18.53	-25.39	-75.82
120	$120^{\bullet+}$	0.100	14.36	-19.67	-75.18

[a] The values for $\Delta G_{HA}(N^{\bullet+})$, $pK_a(NH^+)$ and $E_{red}(N^{\bullet+})$ have been previously reported in our work (Zhao, X.; Hou, Y.-L.; Qian, B.-C.;* Shen, G.-B.* Thermodynamic H-Abstraction Abilities of Nitrogen Centered Radical Cations as Potential HAT Catalysts in Y-H Bond Functionalization. *ACS Omega*, **2024**, *9*, 26708–26718.). [b] $E_{ox}(N) = E_{red}(N^{\bullet+})$. [c] $-\Delta G_{PD}(NH^+) = \Delta G_{PA}(N) = -1.37pK_a(NH^+)$. [d] $\Delta G_{HD}(NH^+) = -\Delta G_{HA}(N^{\bullet+})$.

Thermodynamic graphs of 120 N$^{\bullet+}$ abstracting hydrogen atoms in acetontrile

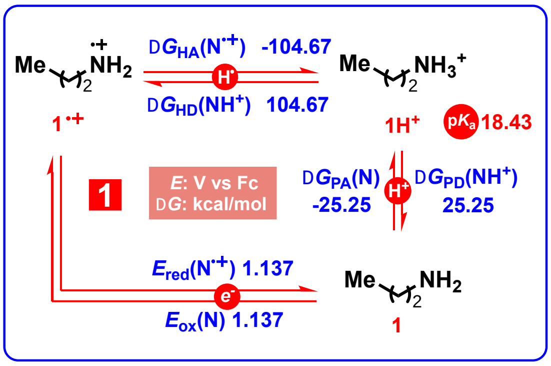

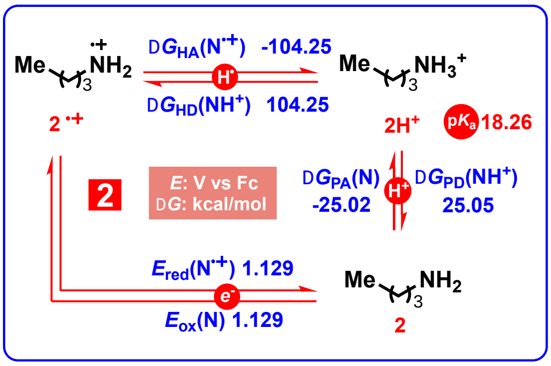

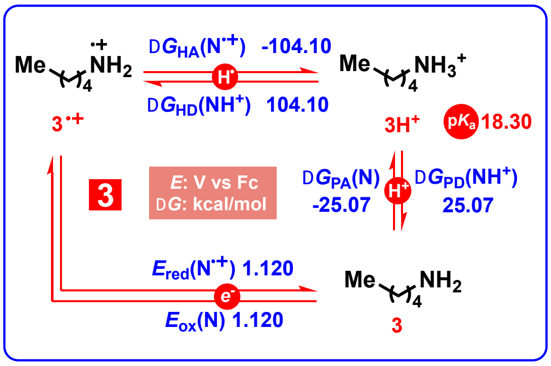

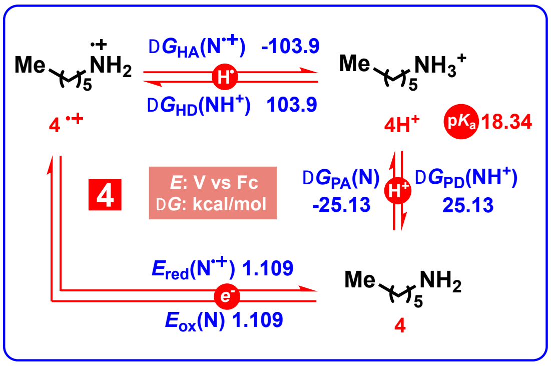

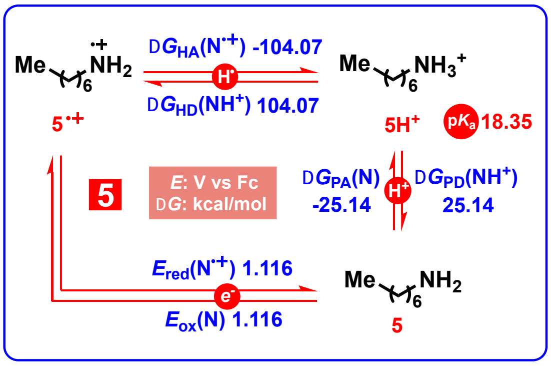

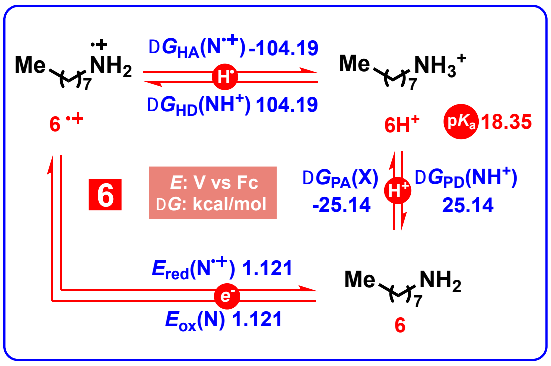

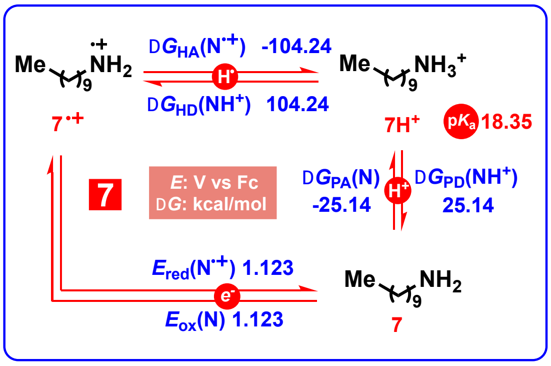

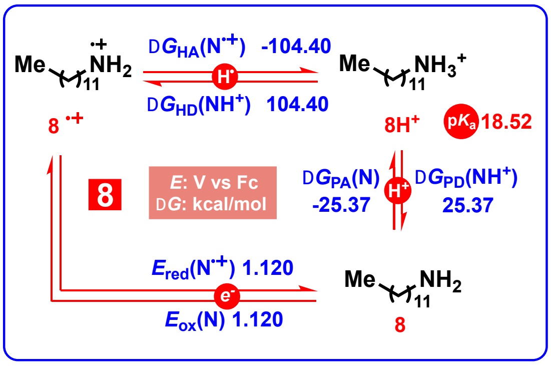

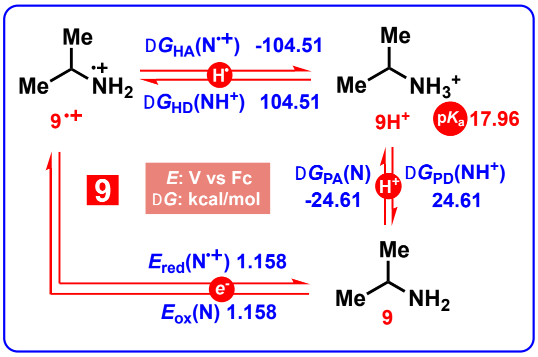
Me
Me
NH2
9•+
DGHA(N•+) -104.51
H•
DGHD(NH+) 104.51
9H+
pKa 17.96
9
E: V vs Fc
DG: kcal/mol
DGPA(N) -24.61
H+
DGPD(NH+) 24.61
Ered(N•+) 1.158
e-
Eox(N) 1.158
9

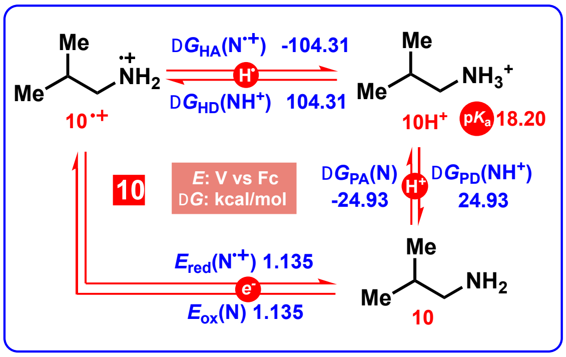
10•+
DGHA(N•+) -104.31
H•
DGHD(NH+) 104.31
10H+
pKa 18.20
10
E: V vs Fc
DG: kcal/mol
DGPA(N) -24.93
H+
DGPD(NH+) 24.93
Ered(N•+) 1.135
e-
Eox(N) 1.135
10

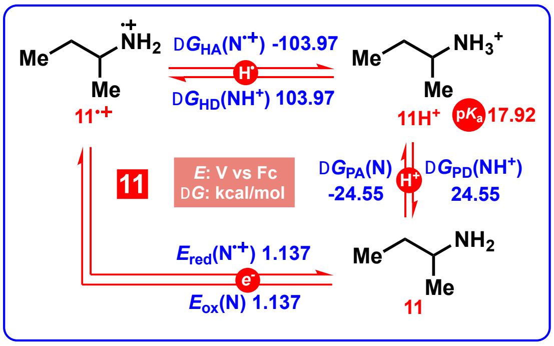
11•+
DGHA(N•+) -103.97
H•
DGHD(NH+) 103.97
11H+
pKa 17.92
11
E: V vs Fc
DG: kcal/mol
DGPA(N) -24.55
H+
DGPD(NH+) 24.55
Ered(N•+) 1.137
e-
Eox(N) 1.137
11

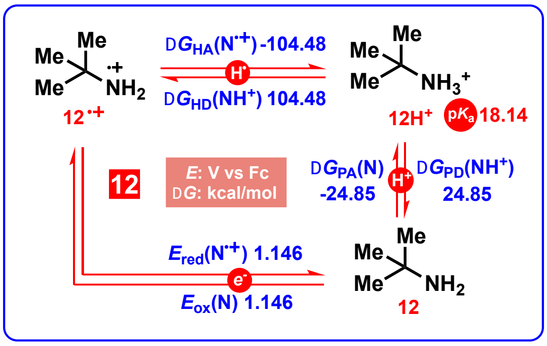
12•+
DGHA(N•+) -104.48
H•
DGHD(NH+) 104.48
12H+
pKa 18.14
12
E: V vs Fc
DG: kcal/mol
DGPA(N) -24.85
H+
DGPD(NH+) 24.85
Ered(N•+) 1.146
e-
Eox(N) 1.146
12

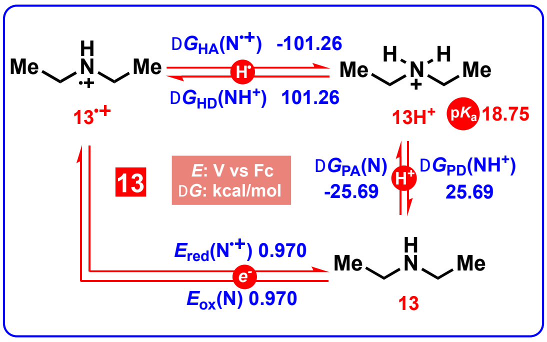
13•+
DGHA(N•+) -101.26
H•
DGHD(NH+) 101.26
13H+
pKa 18.75
13
E: V vs Fc
DG: kcal/mol
DGPA(N) -25.69
H+
DGPD(NH+) 25.69
Ered(N•+) 0.970
e-
Eox(N) 0.970
13

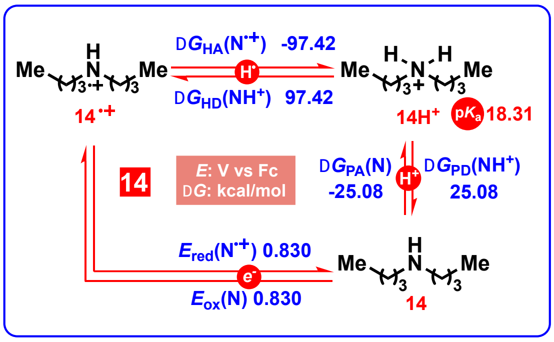
14•+
DGHA(N•+) -97.42
H•
DGHD(NH+) 97.42
14H+
pKa 18.31
14
E: V vs Fc
DG: kcal/mol
DGPA(N) -25.08
H+
DGPD(NH+) 25.08
Ered(N•+) 0.830
e-
Eox(N) 0.830
14

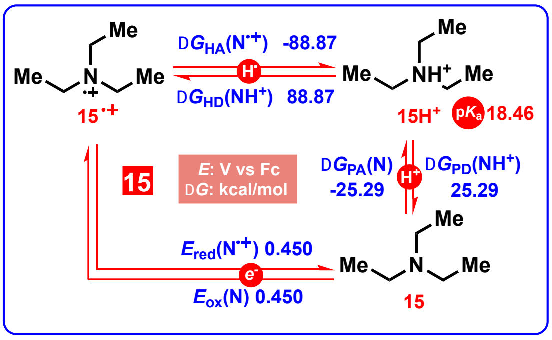
15•+
DGHA(N•+) -88.87
H•
DGHD(NH+) 88.87
15H+
pKa 18.46
15
E: V vs Fc
DG: kcal/mol
DGPA(N) -25.29
H+
DGPD(NH+) 25.29
Ered(N•+) 0.450
e-
Eox(N) 0.450
15

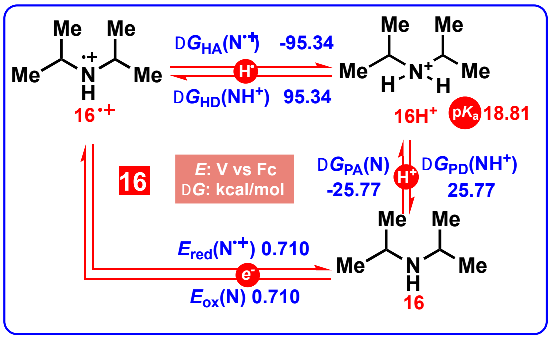
16•+
DGHA(N•+) -95.34
H•
DGHD(NH+) 95.34
16H+
pKa 18.81
16
E: V vs Fc
DG: kcal/mol
DGPA(N) -25.77
H+
DGPD(NH+) 25.77
Ered(N•+) 0.710
e-
Eox(N) 0.710
16

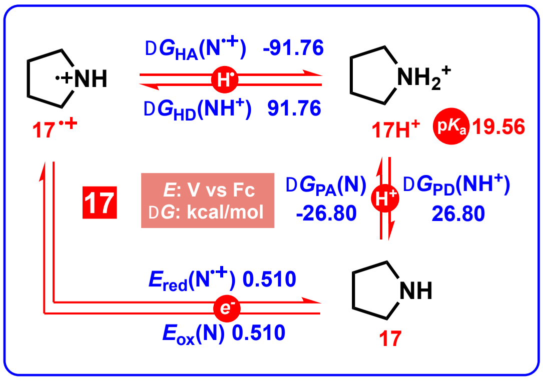
DGHA(N·+) -91.76
DGHD(NH+) 91.76
17·+
17H+ pKa 19.56
17
E: V vs Fc
DG: kcal/mol
DGPA(N) -26.80
DGPD(NH+) 26.80
Ered(N·+) 0.510
Eox(N) 0.510
17

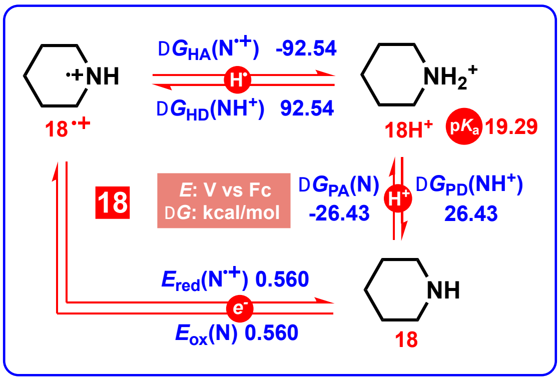
DGHA(N·+) -92.54
DGHD(NH+) 92.54
18·+
18H+ pKa 19.29
18
E: V vs Fc
DG: kcal/mol
DGPA(N) -26.43
DGPD(NH+) 26.43
Ered(N·+) 0.560
Eox(N) 0.560
18

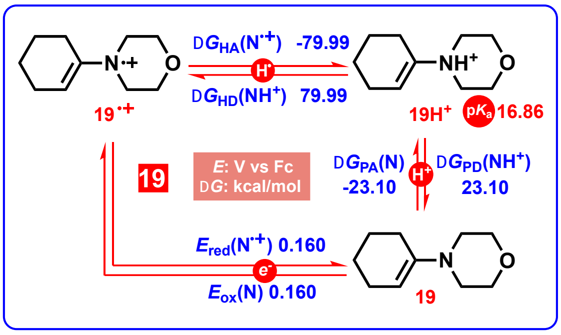
DGHA(N·+) -79.99
DGHD(NH+) 79.99
19·+
19H+ pKa 16.86
19
E: V vs Fc
DG: kcal/mol
DGPA(N) -23.10
DGPD(NH+) 23.10
Ered(N·+) 0.160
Eox(N) 0.160
19

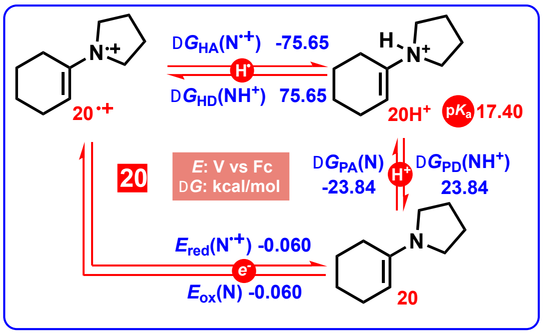
DGHA(N·+) -75.65
DGHD(NH+) 75.65
20·+
20H+ pKa 17.40
20
E: V vs Fc
DG: kcal/mol
DGPA(N) -23.84
DGPD(NH+) 23.84
Ered(N·+) -0.060
Eox(N) -0.060
20

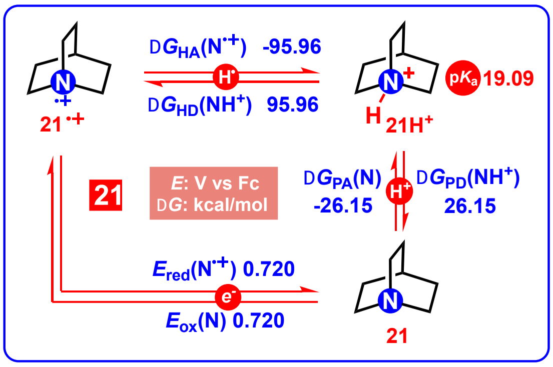
DGHA(N·+) -95.96
DGHD(NH+) 95.96
21·+
21H+ pKa 19.09
21
E: V vs Fc
DG: kcal/mol
DGPA(N) -26.15
DGPD(NH+) 26.15
Ered(N·+) 0.720
Eox(N) 0.720
21

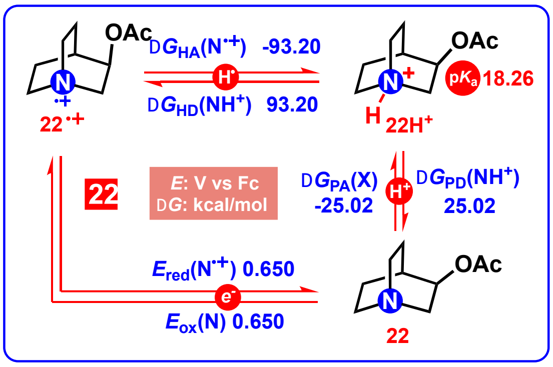
OAc
DGHA(N·+) -93.20
DGHD(NH+) 93.20
22·+
22H+ pKa 18.26
22
E: V vs Fc
DG: kcal/mol
DGPA(X) -25.02
DGPD(NH+) 25.02
Ered(N·+) 0.650
Eox(N) 0.650
22

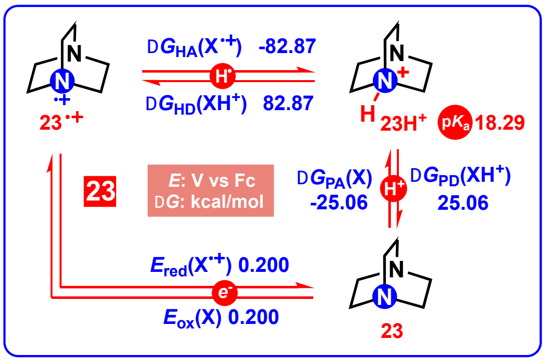
DGHA(X·+) -82.87
DGHD(XH+) 82.87
23·+
23H+ pKa 18.29
23
E: V vs Fc
DG: kcal/mol
DGPA(X) -25.06
DGPD(XH+) 25.06
Ered(X·+) 0.200
Eox(X) 0.200
23

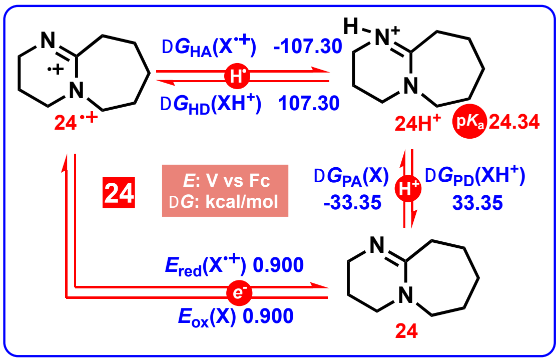
DGHA(X·+) -107.30
DGHD(XH+) 107.30
24·+
24H+ pKa 24.34
24
E: V vs Fc
DG: kcal/mol
DGPA(X) -33.35
DGPD(XH+) 33.35
Ered(X·+) 0.900
Eox(X) 0.900
24

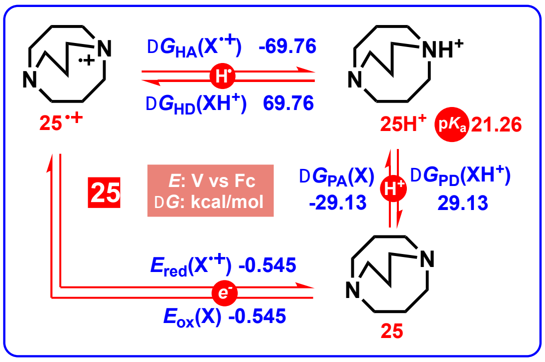
25
E: V vs Fc
DG: kcal/mol
DGHA(X·+) -69.76
DGHD(XH+) 69.76
25·+
25H+ pKa 21.26
DGPA(X) -29.13
DGPD(XH+) 29.13
Ered(X·+) -0.545
Eox(X) -0.545
25

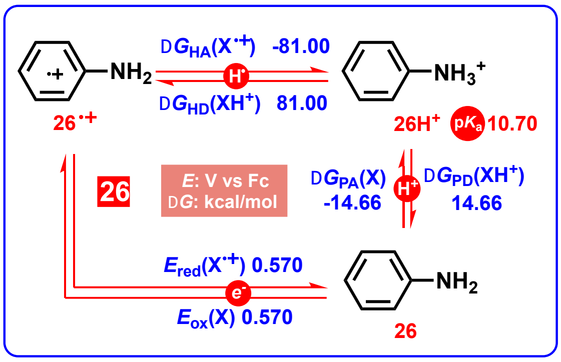
26
E: V vs Fc
DG: kcal/mol
NH2
NH3+
DGHA(X·+) -81.00
DGHD(XH+) 81.00
26·+
26H+ pKa 10.70
DGPA(X) -14.66
DGPD(XH+) 14.66
Ered(X·+) 0.570
Eox(X) 0.570
26

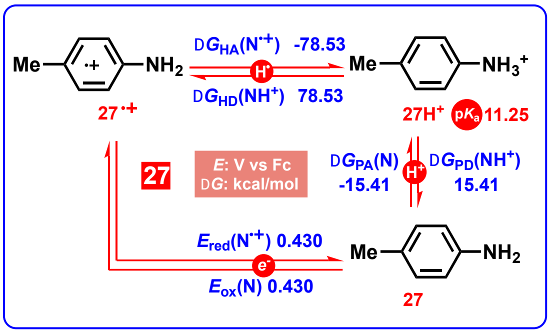
27
E: V vs Fc
DG: kcal/mol
Me
NH2
NH3+
DGHA(N·+) -78.53
DGHD(NH+) 78.53
27·+
27H+ pKa 11.25
DGPA(N) -15.41
DGPD(NH+) 15.41
Ered(N·+) 0.430
Eox(N) 0.430
27

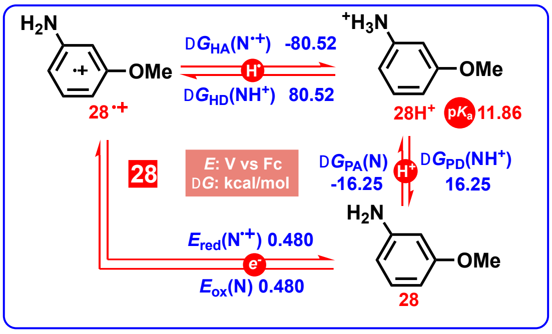
28
E: V vs Fc
DG: kcal/mol
H2N
+H3N
OMe
DGHA(N·+) -80.52
DGHD(NH+) 80.52
28·+
28H+ pKa 11.86
DGPA(N) -16.25
DGPD(NH+) 16.25
Ered(N·+) 0.480
Eox(N) 0.480
28

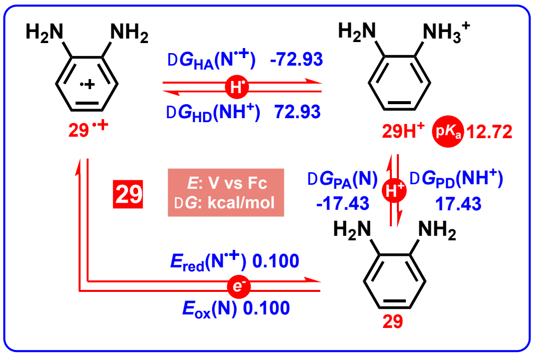
29
E: V vs Fc
DG: kcal/mol
H2N
NH2
NH3+
DGHA(N·+) -72.93
DGHD(NH+) 72.93
29·+
29H+ pKa 12.72
DGPA(N) -17.43
DGPD(NH+) 17.43
Ered(N·+) 0.100
Eox(N) 0.100
29

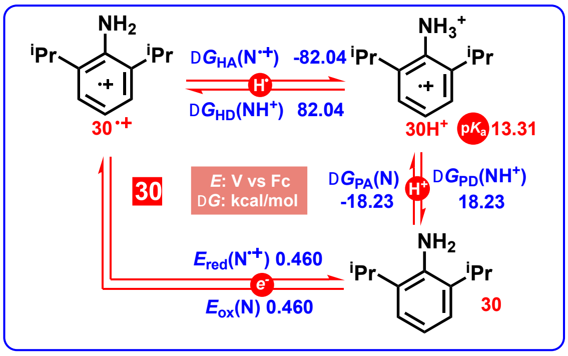
30
E: V vs Fc
DG: kcal/mol
NH2
NH3+
iPr
DGHA(N·+) -82.04
DGHD(NH+) 82.04
30·+
30H+ pKa 13.31
DGPA(N) -18.23
DGPD(NH+) 18.23
Ered(N·+) 0.460
Eox(N) 0.460
30

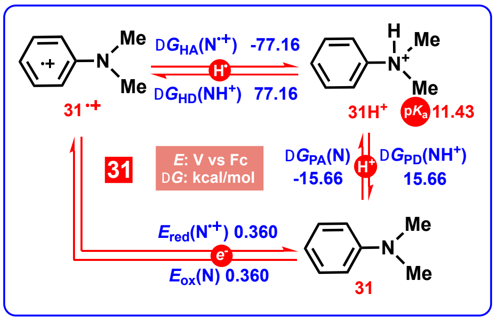
31
E: V vs Fc
DG: kcal/mol
Me
N
H
DGHA(N·+) -77.16
DGHD(NH+) 77.16
31·+
31H+ pKa 11.43
DGPA(N) -15.66
DGPD(NH+) 15.66
Ered(N·+) 0.360
Eox(N) 0.360
31

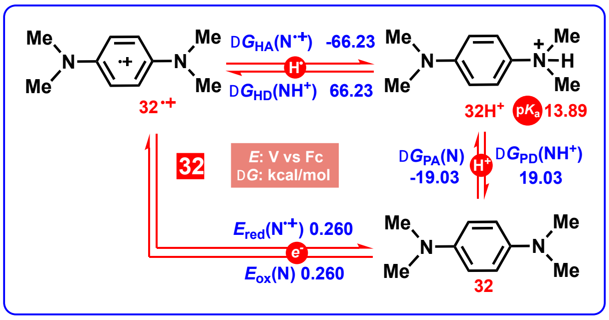
32
E: V vs Fc
DG: kcal/mol
Me
N
H
DGHA(N·+) -66.23
DGHD(NH+) 66.23
32·+
32H+ pKa 13.89
DGPA(N) -19.03
DGPD(NH+) 19.03
Ered(N·+) 0.260
Eox(N) 0.260
32

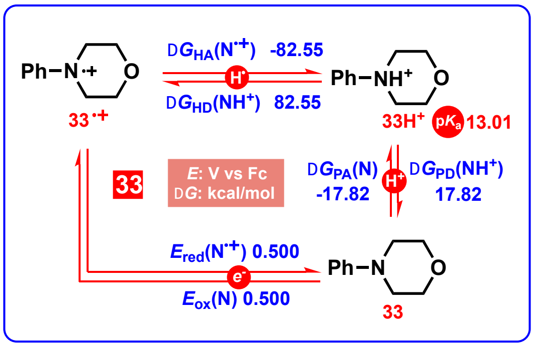
33
E: V vs Fc
DG: kcal/mol
Ph-N
O
Ph-NH+
DGHA(N·+) -82.55
DGHD(NH+) 82.55
33·+
33H+ pKa 13.01
DGPA(N) -17.82
DGPD(NH+) 17.82
Ered(N·+) 0.500
Eox(N) 0.500
33

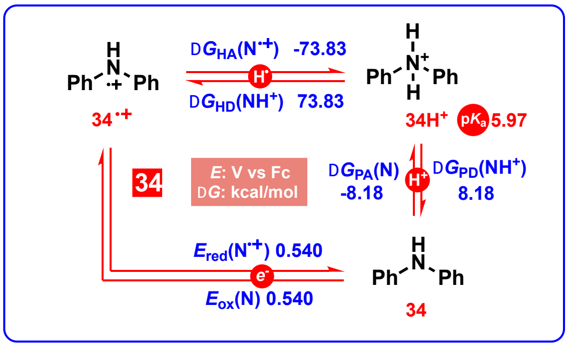
34
E: V vs Fc
DG: kcal/mol
Ph
N
H
DGHA(N·+) -73.83
DGHD(NH+) 73.83
34·+
34H+ pKa 5.97
DGPA(N) -8.18
DGPD(NH+) 8.18
Ered(N·+) 0.540
Eox(N) 0.540
34

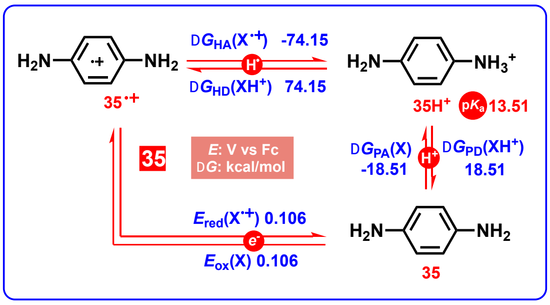
H2N NH2
35·+
DGHA(X·+) -74.15
DGHD(XH+) 74.15
H2N NH3+
35H+ pKa 13.51
35
E: V vs Fc
DG: kcal/mol
DGPA(X) -18.51
DGPD(XH+) 18.51
Ered(X·+) 0.106
Eox(X) 0.106
H2N NH2
35

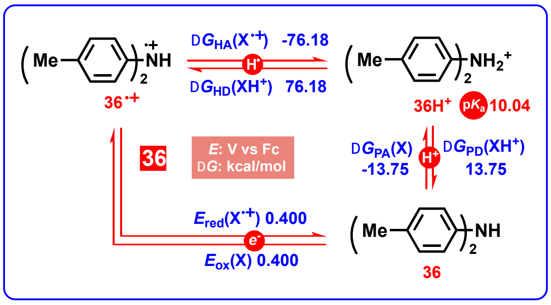
Me NH
36·+
DGHA(X·+) -76.18
DGHD(XH+) 76.18
Me NH2+
36H+ pKa 10.04
36
E: V vs Fc
DG: kcal/mol
DGPA(X) -13.75
DGPD(XH+) 13.75
Ered(X·+) 0.400
Eox(X) 0.400
Me NH
36

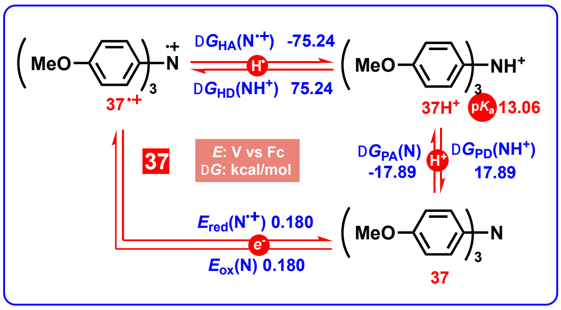
MeO N
37·+
DGHA(N·+) -75.24
DGHD(NH+) 75.24
MeO NH+
37H+ pKa 13.06
37
E: V vs Fc
DG: kcal/mol
DGPA(N) -17.89
DGPD(NH+) 17.89
Ered(N·+) 0.180
Eox(N) 0.180
MeO N
37

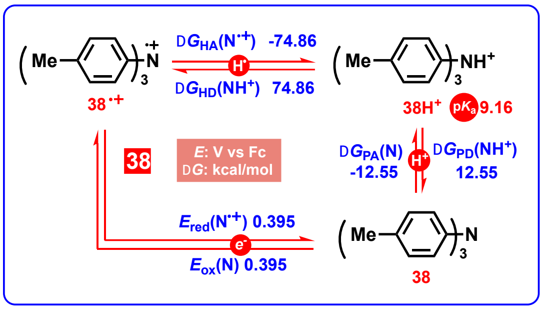
Me N
38·+
DGHA(N·+) -74.86
DGHD(NH+) 74.86
Me NH+
38H+ pKa 9.16
38
E: V vs Fc
DG: kcal/mol
DGPA(N) -12.55
DGPD(NH+) 12.55
Ered(N·+) 0.395
Eox(N) 0.395
Me N
38

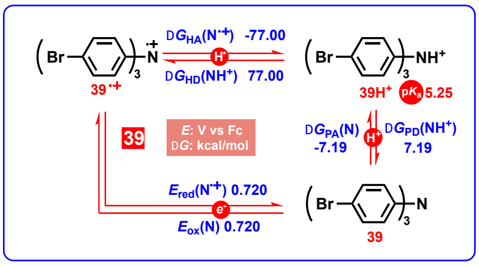
Br N
39·+
DGHA(N·+) -77.00
DGHD(NH+) 77.00
Br NH+
39H+ pKa 5.25
39
E: V vs Fc
DG: kcal/mol
DGPA(N) -7.19
DGPD(NH+) 7.19
Ered(N·+) 0.720
Eox(N) 0.720
Br N
39

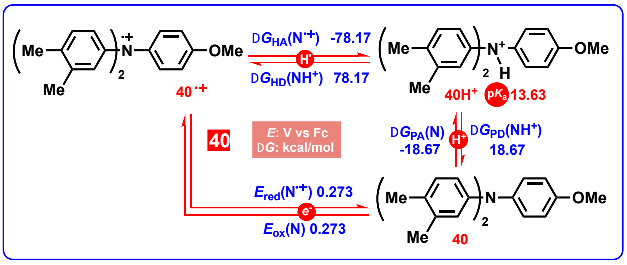
Me Me N OMe
40·+
DGHA(N·+) -78.17
DGHD(NH+) 78.17
Me Me N+ H OMe
40H+ pKa 13.63
40
E: V vs Fc
DG: kcal/mol
DGPA(N) -18.67
DGPD(NH+) 18.67
Ered(N·+) 0.273
Eox(N) 0.273
Me Me N OMe
40

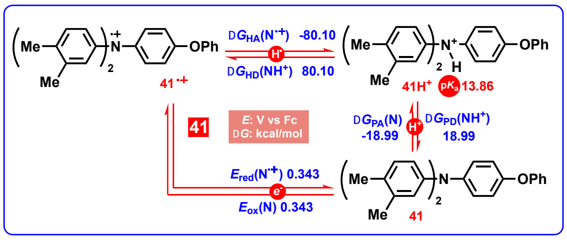
Me Me N OPh
41·+
DGHA(N·+) -80.10
DGHD(NH+) 80.10
Me Me N+ H OPh
41H+ pKa 13.86
41
E: V vs Fc
DG: kcal/mol
DGPA(N) -18.99
DGPD(NH+) 18.99
Ered(N·+) 0.343
Eox(N) 0.343
Me Me N OPh
41

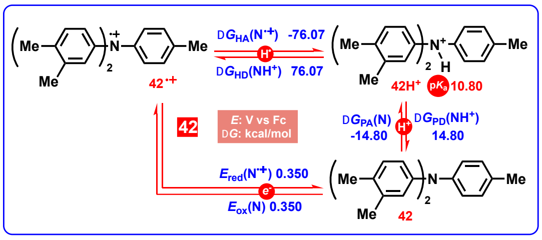
Me Me N Me
42·+
DGHA(N·+) -76.07
DGHD(NH+) 76.07
Me Me N+ H Me
42H+ pKa 10.80
42
E: V vs Fc
DG: kcal/mol
DGPA(N) -14.80
DGPD(NH+) 14.80
Ered(N·+) 0.350
Eox(N) 0.350
Me Me N Me
42

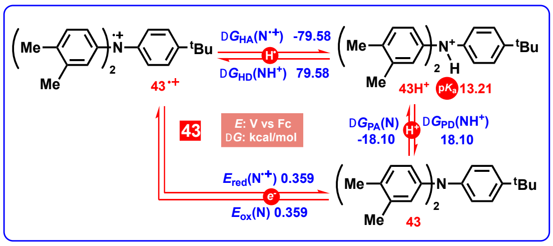
Me Me N tBu
43·+
DGHA(N·+) -79.58
DGHD(NH+) 79.58
Me Me N+ H tBu
43H+ pKa 13.21
43
E: V vs Fc
DG: kcal/mol
DGPA(N) -18.10
DGPD(NH+) 18.10
Ered(N·+) 0.359
Eox(N) 0.359
Me Me N tBu
43

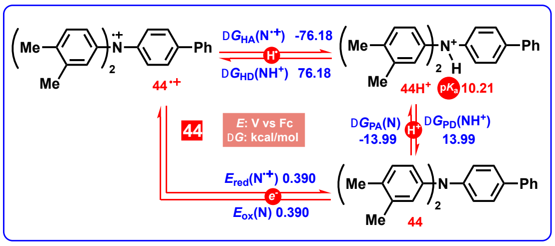
Me Me N Ph
44·+
DGHA(N·+) -76.18
DGHD(NH+) 76.18
Me Me N+ H Ph
44H+ pKa 10.21
44
E: V vs Fc
DG: kcal/mol
DGPA(N) -13.99
DGPD(NH+) 13.99
Ered(N·+) 0.390
Eox(N) 0.390
Me Me N Ph
44

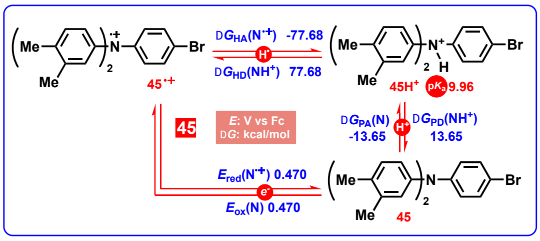
Me Me N Br
45·+
DGHA(N·+) -77.68
DGHD(NH+) 77.68
Me Me N+ H Br
45H+ pKa 9.96
45
E: V vs Fc
DG: kcal/mol
DGPA(N) -13.65
DGPD(NH+) 13.65
Ered(N·+) 0.470
Eox(N) 0.470
Me Me N Br
45

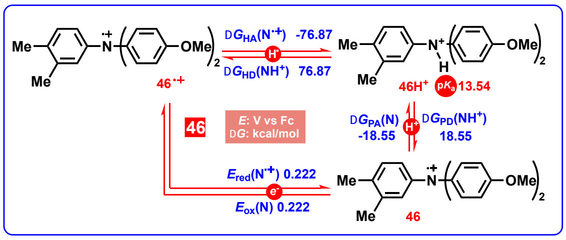
Me Me N OMe
46·+
DGHA(N·+) -76.87
DGHD(NH+) 76.87
Me Me N+ H OMe
46H+ pKa 13.54
46
E: V vs Fc
DG: kcal/mol
DGPA(N) -18.55
DGPD(NH+) 18.55
Ered(N·+) 0.222
Eox(N) 0.222
Me Me N OMe
46

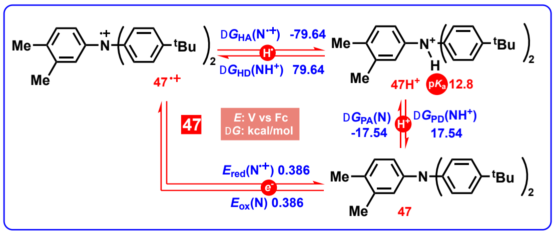
47
DGHA(N•+) -79.64
DGHD(NH+) 79.64
47•+
47H+ pKa 12.8
E: V vs Fc
DG: kcal/mol
DGPA(N) -17.54
DGPD(NH+) 17.54
Ered(N•+) 0.386
Eox(N) 0.386
47

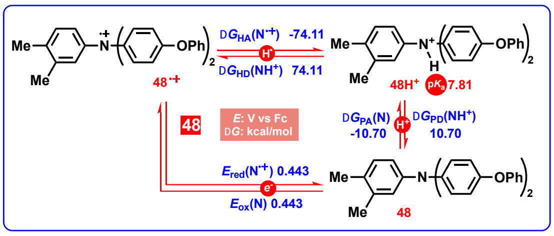
48
DGHA(N•+) -74.11
DGHD(NH+) 74.11
48•+
48H+ pKa 7.81
E: V vs Fc
DG: kcal/mol
DGPA(N) -10.70
DGPD(NH+) 10.70
Ered(N•+) 0.443
Eox(N) 0.443
48

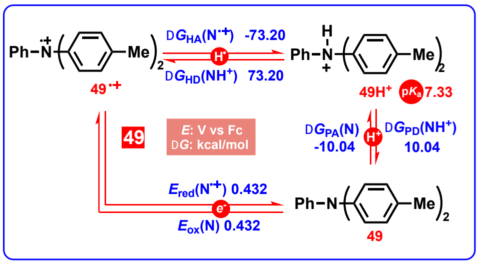
49
DGHA(N•+) -73.20
DGHD(NH+) 73.20
49•+
49H+ pKa 7.33
E: V vs Fc
DG: kcal/mol
DGPA(N) -10.04
DGPD(NH+) 10.04
Ered(N•+) 0.432
Eox(N) 0.432
49

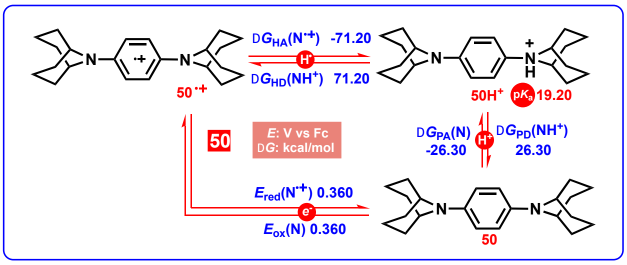
50
DGHA(N•+) -71.20
DGHD(NH+) 71.20
50•+
50H+ pKa 19.20
E: V vs Fc
DG: kcal/mol
DGPA(N) -26.30
DGPD(NH+) 26.30
Ered(N•+) 0.360
Eox(N) 0.360
50

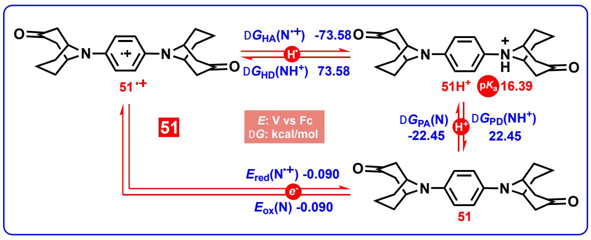
51
DGHA(N•+) -73.58
DGHD(NH+) 73.58
51•+
51H+ pKa 16.39
E: V vs Fc
DG: kcal/mol
DGPA(N) -22.45
DGPD(NH+) 22.45
Ered(N•+) -0.090
Eox(N) -0.090
51

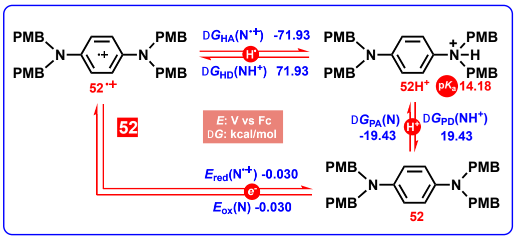
52
DGHA(N•+) -71.93
DGHD(NH+) 71.93
52•+
52H+ pKa 14.18
E: V vs Fc
DG: kcal/mol
DGPA(N) -19.43
DGPD(NH+) 19.43
Ered(N•+) -0.030
Eox(N) -0.030
52

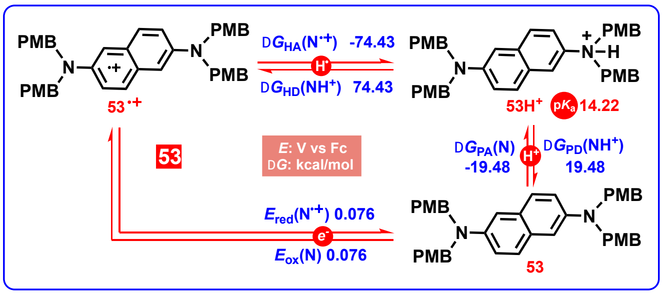
53
DGHA(N•+) -74.43
DGHD(NH+) 74.43
53•+
53H+ pKa 14.22
E: V vs Fc
DG: kcal/mol
DGPA(N) -19.48
DGPD(NH+) 19.48
Ered(N•+) 0.076
Eox(N) 0.076
53

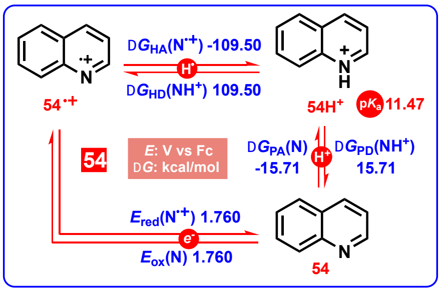
54
DGHA(N•+) -109.50
DGHD(NH+) 109.50
54•+
54H+ pKa 11.47
E: V vs Fc
DG: kcal/mol
DGPA(N) -15.71
DGPD(NH+) 15.71
Ered(N•+) 1.760
Eox(N) 1.760
54

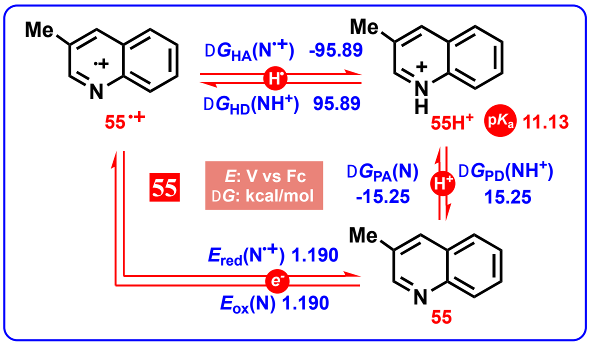
55
DGHA(N•+) -95.89
DGHD(NH+) 95.89
55•+
55H+ pKa 11.13
E: V vs Fc
DG: kcal/mol
DGPA(N) -15.25
DGPD(NH+) 15.25
Ered(N•+) 1.190
Eox(N) 1.190
55

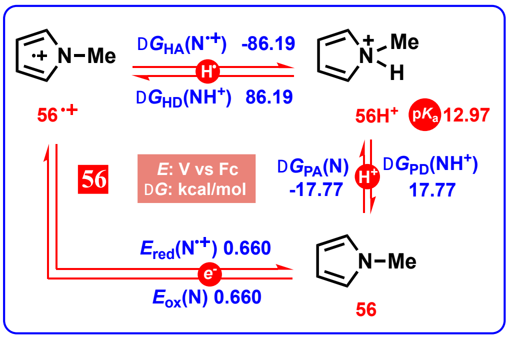
56
DGHA(N•+) -86.19
DGHD(NH+) 86.19
56•+
56H+ pKa 12.97
E: V vs Fc
DG: kcal/mol
DGPA(N) -17.77
DGPD(NH+) 17.77
Ered(N•+) 0.660
Eox(N) 0.660
56

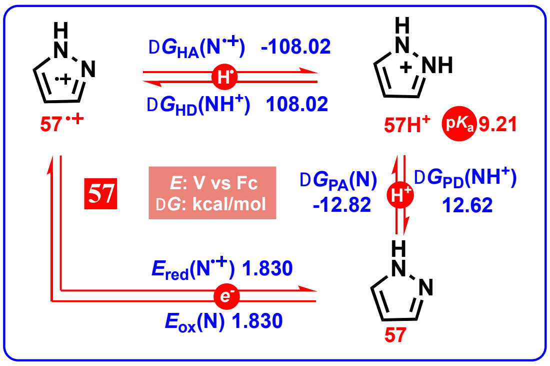
DGHA(N•+) -108.02
DGHD(NH+) 108.02
57•+
57H+ pKa 9.21
57
E: V vs Fc
DG: kcal/mol
DGPA(N) -12.82
DGPD(NH+) 12.62
Ered(N•+) 1.830
Eox(N) 1.830
57

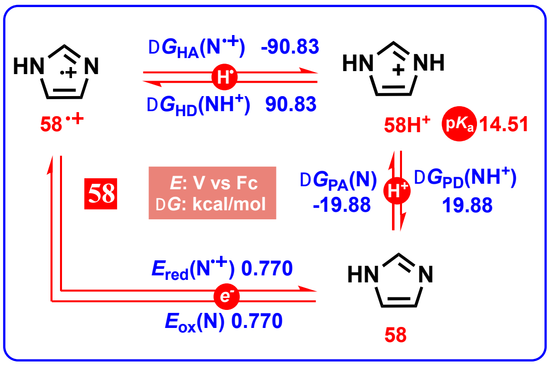
DGHA(N•+) -90.83
DGHD(NH+) 90.83
58•+
58H+ pKa 14.51
58
E: V vs Fc
DG: kcal/mol
DGPA(N) -19.88
DGPD(NH+) 19.88
Ered(N•+) 0.770
Eox(N) 0.770
58

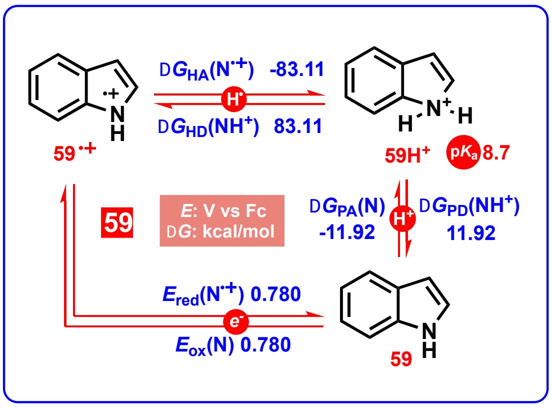
DGHA(N•+) -83.11
DGHD(NH+) 83.11
59•+
59H+ pKa 8.7
59
E: V vs Fc
DG: kcal/mol
DGPA(N) -11.92
DGPD(NH+) 11.92
Ered(N•+) 0.780
Eox(N) 0.780
59

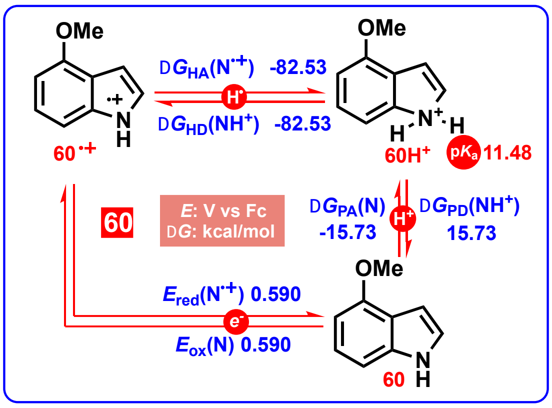
DGHA(N•+) -82.53
DGHD(NH+) -82.53
60•+
60H+ pKa 11.48
60
E: V vs Fc
DG: kcal/mol
DGPA(N) -15.73
DGPD(NH+) 15.73
Ered(N•+) 0.590
Eox(N) 0.590
60

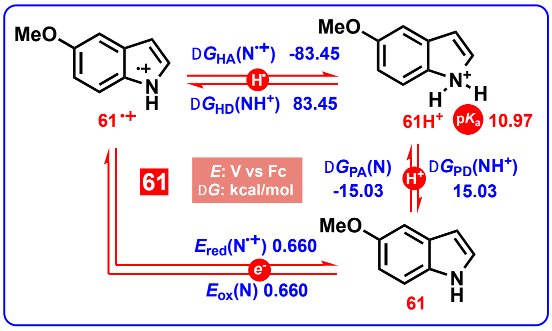
DGHA(N•+) -83.45
DGHD(NH+) 83.45
61•+
61H+ pKa 10.97
61
E: V vs Fc
DG: kcal/mol
DGPA(N) -15.03
DGPD(NH+) 15.03
Ered(N•+) 0.660
Eox(N) 0.660
61

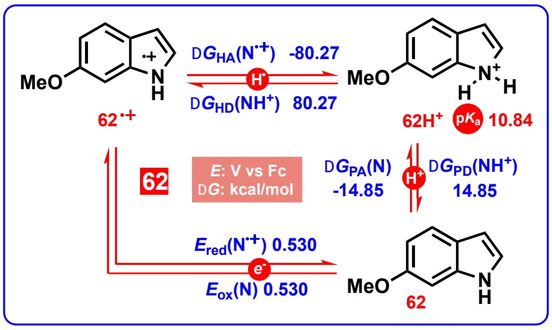
DGHA(N•+) -80.27
DGHD(NH+) 80.27
62•+
62H+ pKa 10.84
62
E: V vs Fc
DG: kcal/mol
DGPA(N) -14.85
DGPD(NH+) 14.85
Ered(N•+) 0.530
Eox(N) 0.530
62

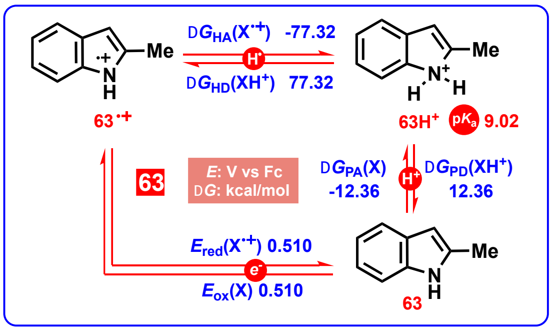
DGHA(X•+) -77.32
DGHD(XH+) 77.32
63•+
63H+ pKa 9.02
63
E: V vs Fc
DG: kcal/mol
DGPA(X) -12.36
DGPD(XH+) 12.36
Ered(X•+) 0.510
Eox(X) 0.510
63

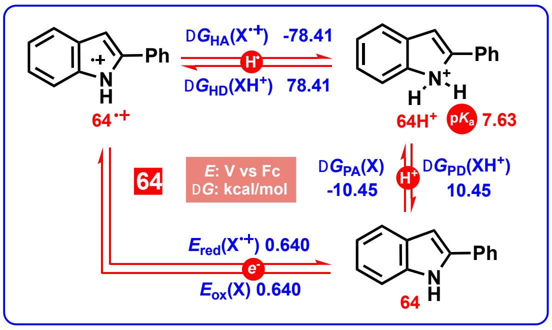
DGHA(X•+) -78.41
DGHD(XH+) 78.41
64•+
64H+ pKa 7.63
64
E: V vs Fc
DG: kcal/mol
DGPA(X) -10.45
DGPD(XH+) 10.45
Ered(X•+) 0.640
Eox(X) 0.640
64

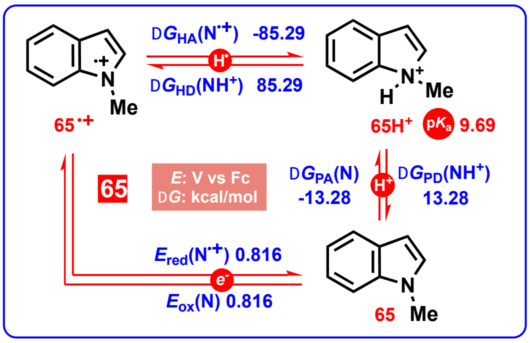
DGHA(N•+) -85.29
DGHD(NH+) 85.29
65•+
65H+ pKa 9.69
65
E: V vs Fc
DG: kcal/mol
DGPA(N) -13.28
DGPD(NH+) 13.28
Ered(N•+) 0.816
Eox(N) 0.816
65

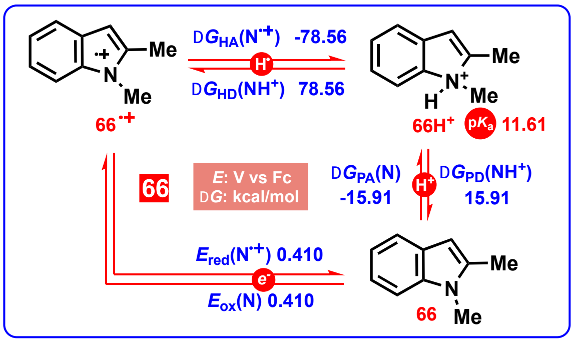
DGHA(N•+) -78.56
DGHD(NH+) 78.56
66•+
66H+ pKa 11.61
66
E: V vs Fc
DG: kcal/mol
DGPA(N) -15.91
DGPD(NH+) 15.91
Ered(N•+) 0.410
Eox(N) 0.410
66

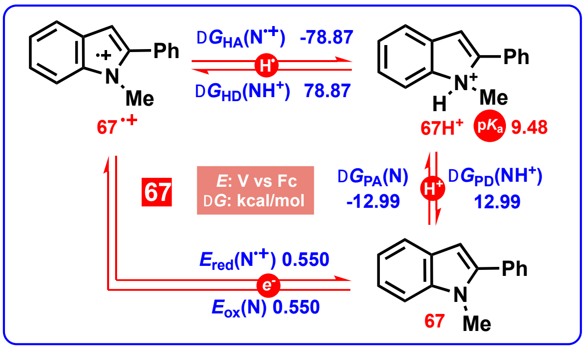
DGHA(N•+) -78.87
DGHD(NH+) 78.87
67•+
67H+ pKa 9.48
67
E: V vs Fc
DG: kcal/mol
DGPA(N) -12.99
DGPD(NH+) 12.99
Ered(N•+) 0.550
Eox(N) 0.550
67

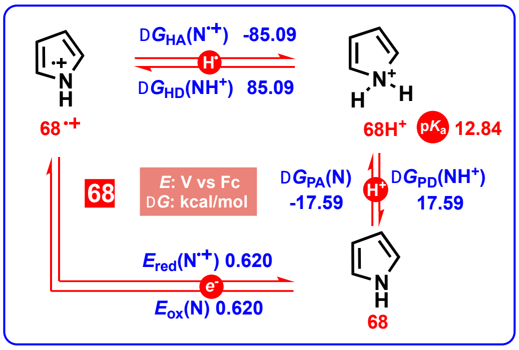
DGHA(N•+) -85.09
DGHD(NH+) 85.09
68•+
68H+ pKa 12.84
68
E: V vs Fc
DG: kcal/mol
DGPA(N) -17.59
DGPD(NH+) 17.59
Ered(N•+) 0.620
Eox(N) 0.620
68

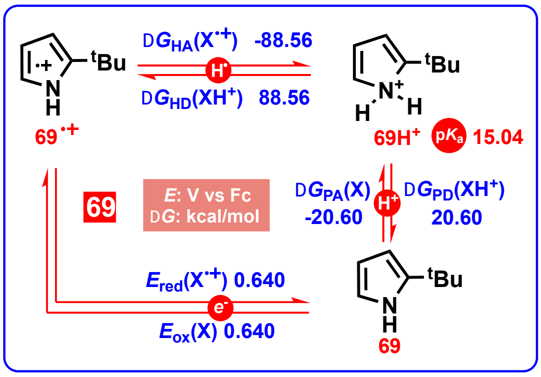
DGHA(X•+) -88.56
DGHD(XH+) 88.56
69•+
69H+ pKa 15.04
69
E: V vs Fc
DG: kcal/mol
DGPA(X) -20.60
DGPD(XH+) 20.60
Ered(X•+) 0.640
Eox(X) 0.640
69

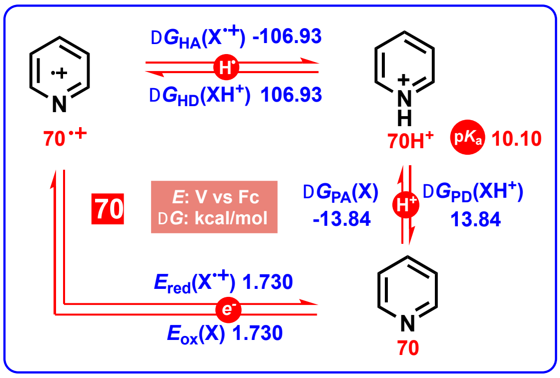
DGHA(X•+) -106.93
DGHD(XH+) 106.93
70•+
70H+ pKa 10.10
70
E: V vs Fc
DG: kcal/mol
DGPA(X) -13.84
DGPD(XH+) 13.84
Ered(X•+) 1.730
Eox(X) 1.730
70

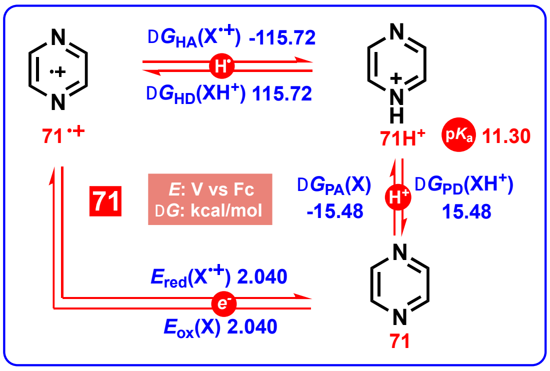
DGHA(X•+) -115.72
DGHD(XH+) 115.72
71•+
71H+ pKa 11.30
71
E: V vs Fc
DG: kcal/mol
DGPA(X) -15.48
DGPD(XH+) 15.48
Ered(X•+) 2.040
Eox(X) 2.040
71

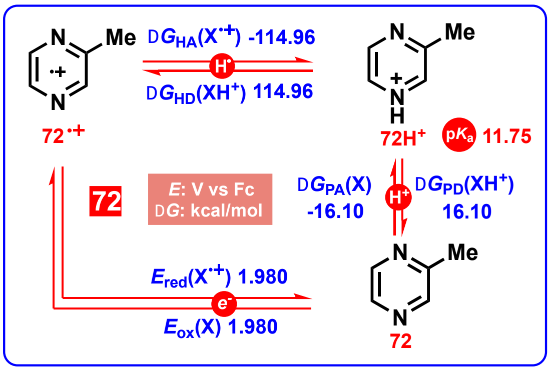
DGHA(X•+) -114.96
DGHD(XH+) 114.96
72•+
72H+ pKa 11.75
72
E: V vs Fc
DG: kcal/mol
DGPA(X) -16.10
DGPD(XH+) 16.10
Ered(X•+) 1.980
Eox(X) 1.980
72

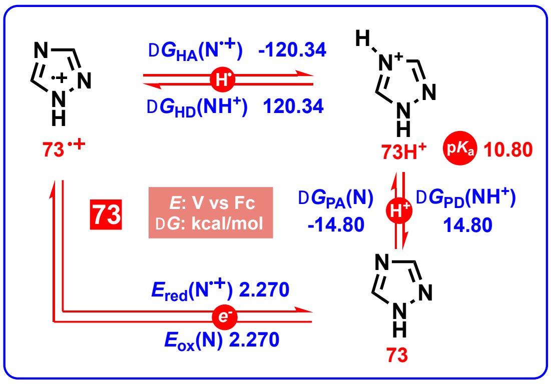
DGHA(N•+) -120.34
DGHD(NH+) 120.34
73•+
73H+ pKa 10.80
73
E: V vs Fc
DG: kcal/mol
DGPA(N) -14.80
DGPD(NH+) 14.80
Ered(N•+) 2.270
Eox(N) 2.270
73

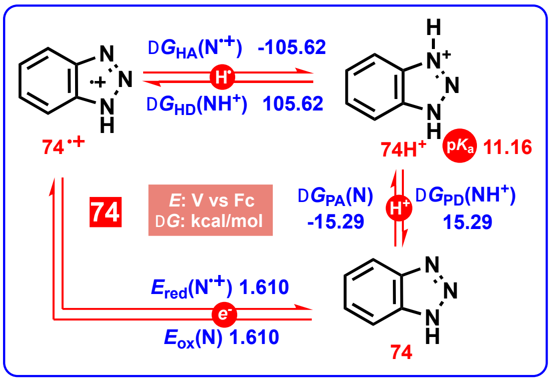
DGHA(N•+) -105.62
DGHD(NH+) 105.62
74•+
74H+ pKa 11.16
74
E: V vs Fc
DG: kcal/mol
DGPA(N) -15.29
DGPD(NH+) 15.29
Ered(N•+) 1.610
Eox(N) 1.610
74

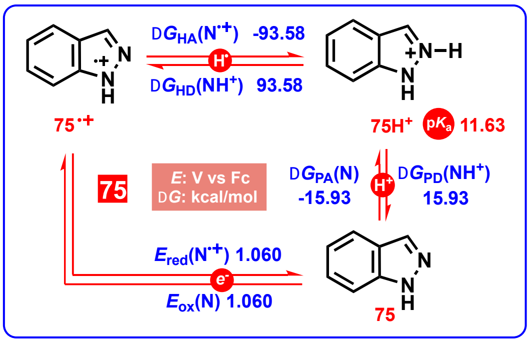
DG_HA(N•+) -93.58
DG_HD(NH+) 93.58
75•+
75H+ pKa 11.63
75
E: V vs Fc
DG: kcal/mol
DG_PA(N) -15.93
DG_PD(NH+) 15.93
E_red(N•+) 1.060
E_ox(N) 1.060
75

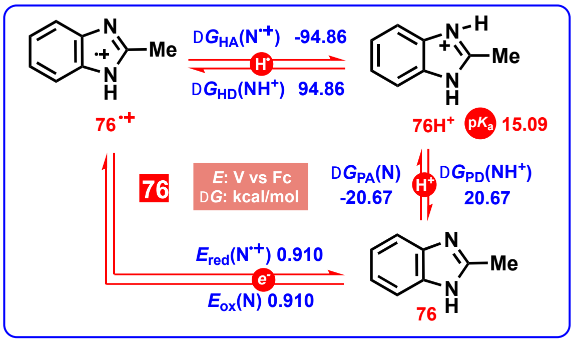
Me
DG_HA(N•+) -94.86
DG_HD(NH+) 94.86
76•+
76H+ pKa 15.09
76
E: V vs Fc
DG: kcal/mol
DG_PA(N) -20.67
DG_PD(NH+) 20.67
E_red(N•+) 0.910
E_ox(N) 0.910
76

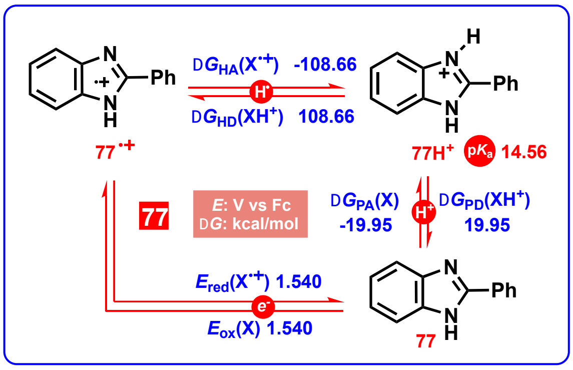
Ph
DG_HA(X•+) -108.66
DG_HD(XH+) 108.66
77•+
77H+ pKa 14.56
77
E: V vs Fc
DG: kcal/mol
DG_PA(X) -19.95
DG_PD(XH+) 19.95
E_red(X•+) 1.540
E_ox(X) 1.540
77

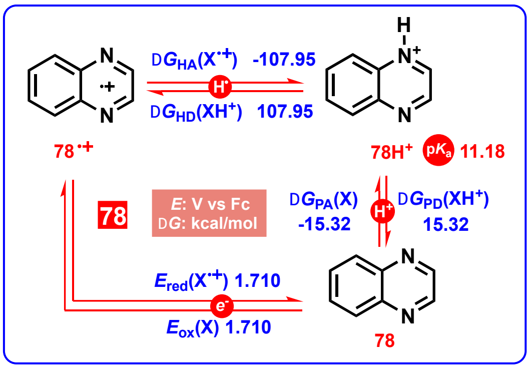
DG_HA(X•+) -107.95
DG_HD(XH+) 107.95
78•+
78H+ pKa 11.18
78
E: V vs Fc
DG: kcal/mol
DG_PA(X) -15.32
DG_PD(XH+) 15.32
E_red(X•+) 1.710
E_ox(X) 1.710
78

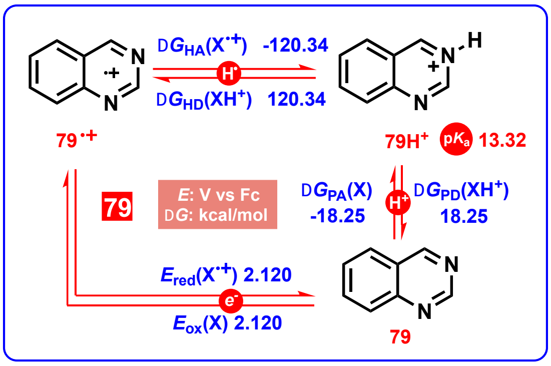
DG_HA(X•+) -120.34
DG_HD(XH+) 120.34
79•+
79H+ pKa 13.32
79
E: V vs Fc
DG: kcal/mol
DG_PA(X) -18.25
DG_PD(XH+) 18.25
E_red(X•+) 2.120
E_ox(X) 2.120
79

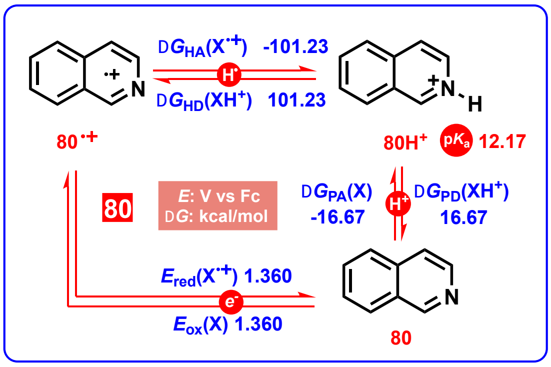
DG_HA(X•+) -101.23
DG_HD(XH+) 101.23
80•+
80H+ pKa 12.17
80
E: V vs Fc
DG: kcal/mol
DG_PA(X) -16.67
DG_PD(XH+) 16.67
E_red(X•+) 1.360
E_ox(X) 1.360
80

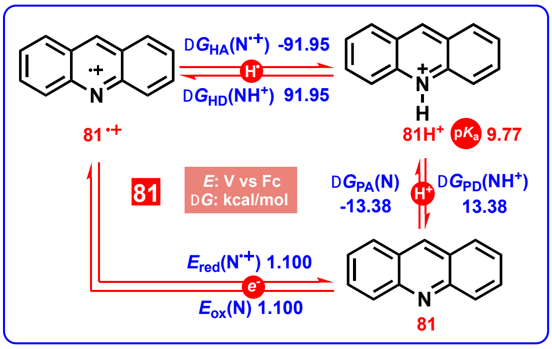
DG_HA(N•+) -91.95
DG_HD(NH+) 91.95
81•+
81H+ pKa 9.77
81
E: V vs Fc
DG: kcal/mol
DG_PA(N) -13.38
DG_PD(NH+) 13.38
E_red(N•+) 1.100
E_ox(N) 1.100
81

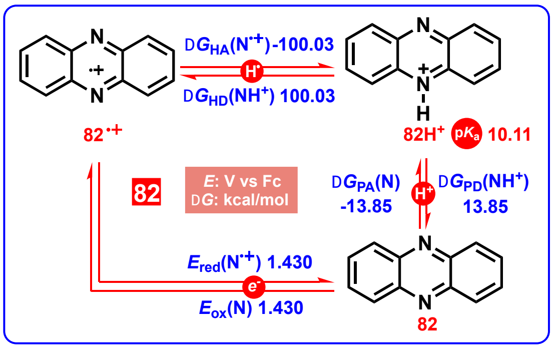
DG_HA(N•+)-100.03
DG_HD(NH+) 100.03
82•+
82H+ pKa 10.11
82
E: V vs Fc
DG: kcal/mol
DG_PA(N) -13.85
DG_PD(NH+) 13.85
E_red(N•+) 1.430
E_ox(N) 1.430
82

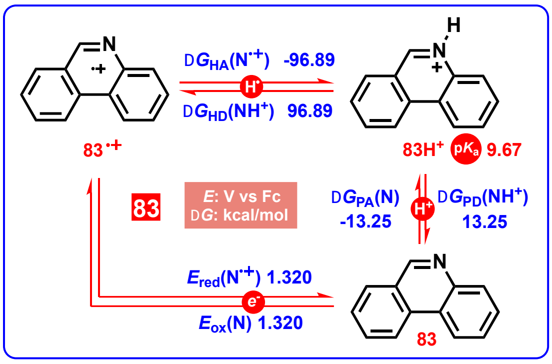
DGHA(N•+) -96.89
DGHD(NH+) 96.89
83•+
83H+ pKa 9.67
83
E: V vs Fc
DG: kcal/mol
DGPA(N) -13.25
DGPD(NH+) 13.25
Ered(N•+) 1.320
Eox(N) 1.320
83

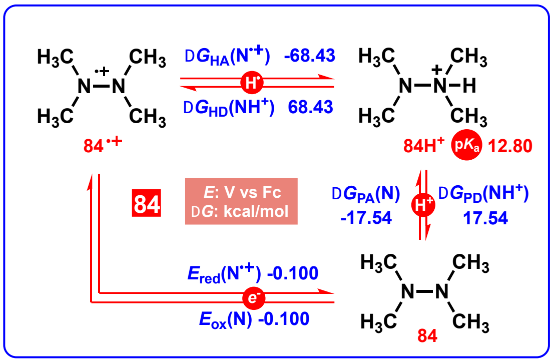
DGHA(N•+) -68.43
DGHD(NH+) 68.43
84•+
84H+ pKa 12.80
84
E: V vs Fc
DG: kcal/mol
DGPA(N) -17.54
DGPD(NH+) 17.54
Ered(N•+) -0.100
Eox(N) -0.100
84

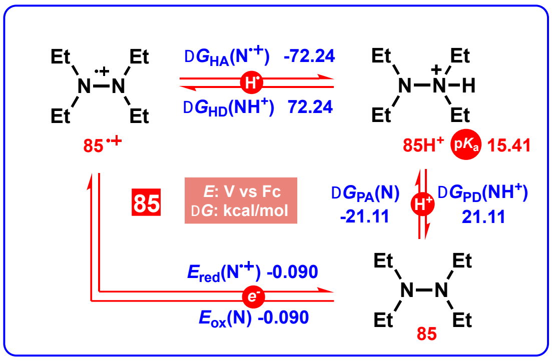
DGHA(N•+) -72.24
DGHD(NH+) 72.24
85•+
85H+ pKa 15.41
85
E: V vs Fc
DG: kcal/mol
DGPA(N) -21.11
DGPD(NH+) 21.11
Ered(N•+) -0.090
Eox(N) -0.090
85

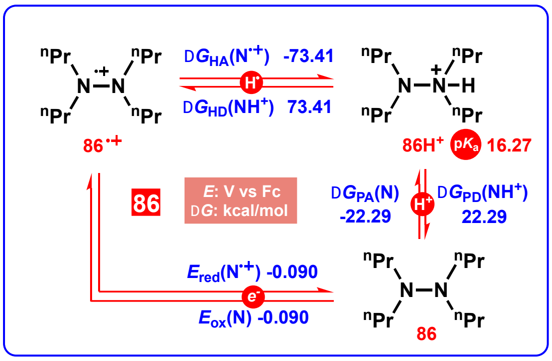
DGHA(N•+) -73.41
DGHD(NH+) 73.41
86•+
86H+ pKa 16.27
86
E: V vs Fc
DG: kcal/mol
DGPA(N) -22.29
DGPD(NH+) 22.29
Ered(N•+) -0.090
Eox(N) -0.090
86

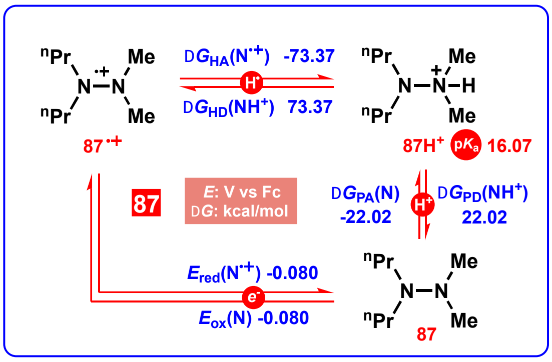
DGHA(N•+) -73.37
DGHD(NH+) 73.37
87•+
87H+ pKa 16.07
87
E: V vs Fc
DG: kcal/mol
DGPA(N) -22.02
DGPD(NH+) 22.02
Ered(N•+) -0.080
Eox(N) -0.080
87

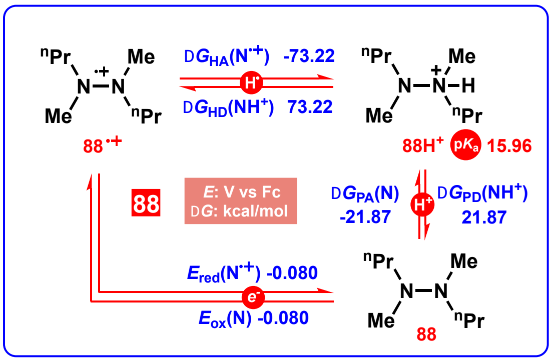
DGHA(N•+) -73.22
DGHD(NH+) 73.22
88•+
88H+ pKa 15.96
88
E: V vs Fc
DG: kcal/mol
DGPA(N) -21.87
DGPD(NH+) 21.87
Ered(N•+) -0.080
Eox(N) -0.080
88

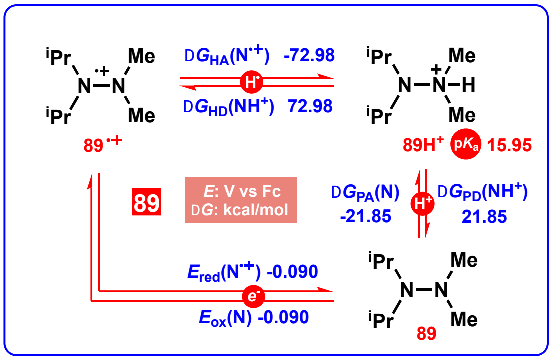
DGHA(N•+) -72.98
DGHD(NH+) 72.98
89•+
89H+ pKa 15.95
89
E: V vs Fc
DG: kcal/mol
DGPA(N) -21.85
DGPD(NH+) 21.85
Ered(N•+) -0.090
Eox(N) -0.090
89

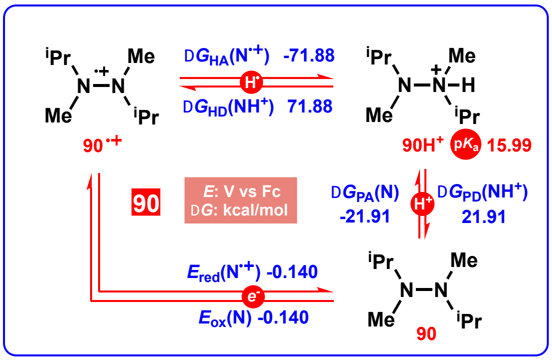
DGHA(N•+) -71.88
DGHD(NH+) 71.88
90•+
90H+ pKa 15.99
90
E: V vs Fc
DG: kcal/mol
DGPA(N) -21.91
DGPD(NH+) 21.91
Ered(N•+) -0.140
Eox(N) -0.140
90

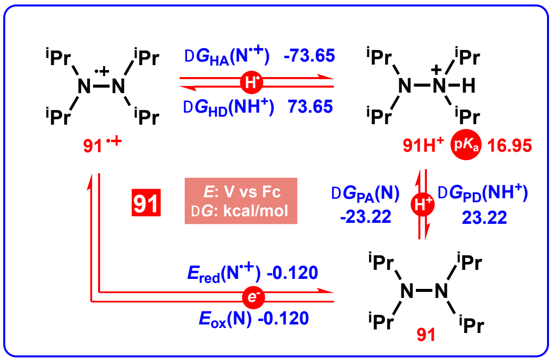

91
E: V vs Fc
DG: kcal/mol
DGHA(N•+) -73.65
DGHD(NH+) 73.65
91H+ pKa 16.95
DGPA(N) -23.22
DGPD(NH+) 23.22
Ered(N•+) -0.120
Eox(N) -0.120
91•+
91

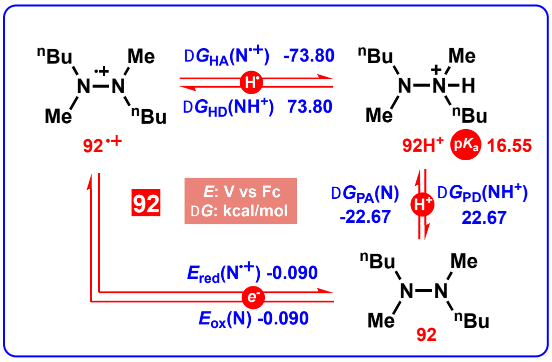

92
E: V vs Fc
DG: kcal/mol
DGHA(N•+) -73.80
DGHD(NH+) 73.80
92H+ pKa 16.55
DGPA(N) -22.67
DGPD(NH+) 22.67
Ered(N•+) -0.090
Eox(N) -0.090
92•+
92

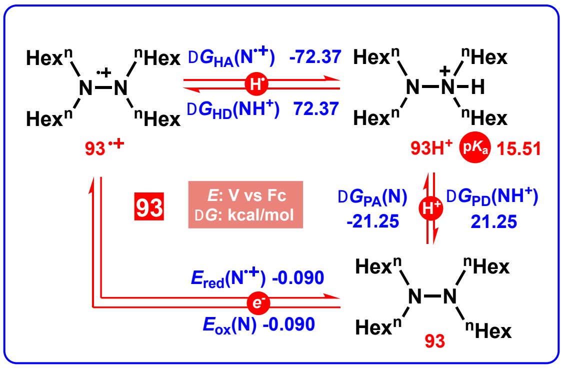

93
E: V vs Fc
DG: kcal/mol
DGHA(N•+) -72.37
DGHD(NH+) 72.37
93H+ pKa 15.51
DGPA(N) -21.25
DGPD(NH+) 21.25
Ered(N•+) -0.090
Eox(N) -0.090
93•+
93

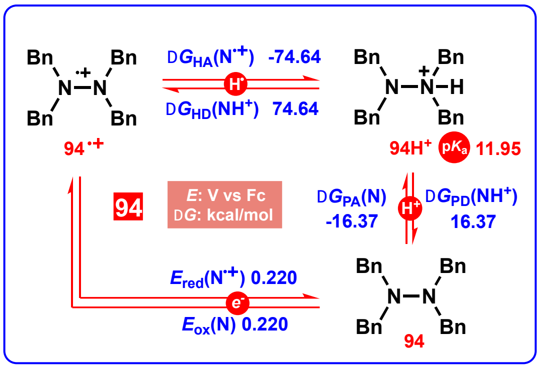

94
E: V vs Fc
DG: kcal/mol
DGHA(N•+) -74.64
DGHD(NH+) 74.64
94H+ pKa 11.95
DGPA(N) -16.37
DGPD(NH+) 16.37
Ered(N•+) 0.220
Eox(N) 0.220
94•+
94

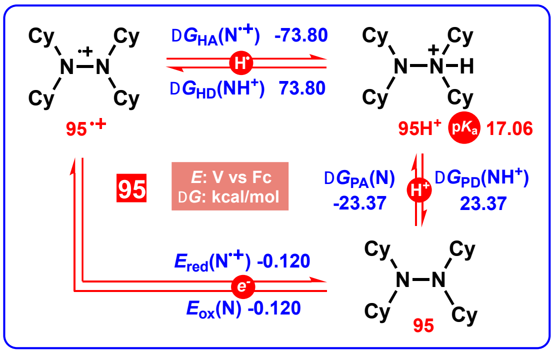

95
E: V vs Fc
DG: kcal/mol
DGHA(N•+) -73.80
DGHD(NH+) 73.80
95H+ pKa 17.06
DGPA(N) -23.37
DGPD(NH+) 23.37
Ered(N•+) -0.120
Eox(N) -0.120
95•+
95

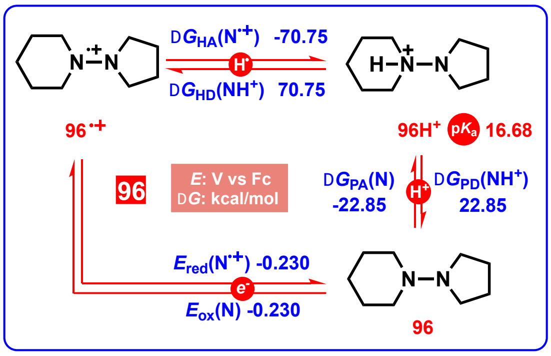

96
E: V vs Fc
DG: kcal/mol
DGHA(N•+) -70.75
DGHD(NH+) 70.75
96H+ pKa 16.68
DGPA(N) -22.85
DGPD(NH+) 22.85
Ered(N•+) -0.230
Eox(N) -0.230
96•+
96

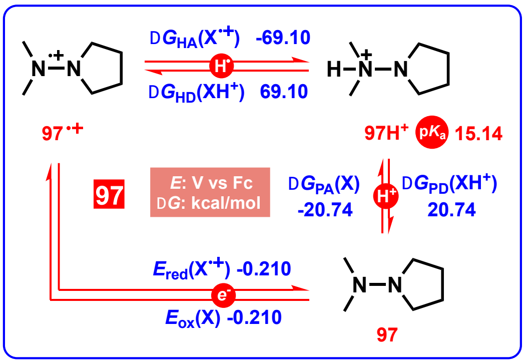

97
E: V vs Fc
DG: kcal/mol
DGHA(X•+) -69.10
DGHD(XH+) 69.10
97H+ pKa 15.14
DGPA(X) -20.74
DGPD(XH+) 20.74
Ered(X•+) -0.210
Eox(X) -0.210
97•+
97

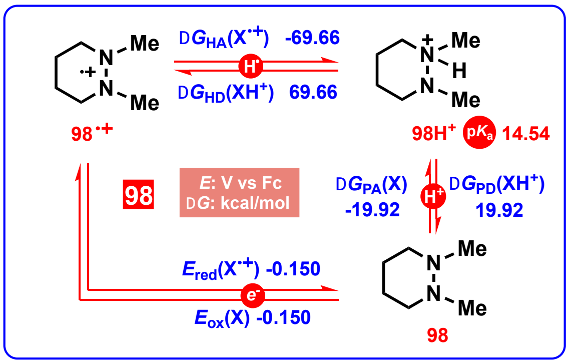

98
E: V vs Fc
DG: kcal/mol
DGHA(X•+) -69.66
DGHD(XH+) 69.66
98H+ pKa 14.54
DGPA(X) -19.92
DGPD(XH+) 19.92
Ered(X•+) -0.150
Eox(X) -0.150
98•+
98

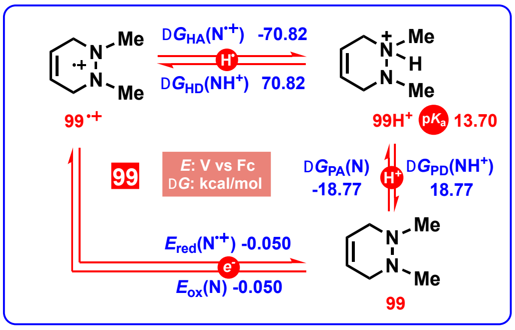

99
E: V vs Fc
DG: kcal/mol
DGHA(N•+) -70.82
DGHD(NH+) 70.82
99H+ pKa 13.70
DGPA(N) -18.77
DGPD(NH+) 18.77
Ered(N•+) -0.050
Eox(N) -0.050
99•+
99

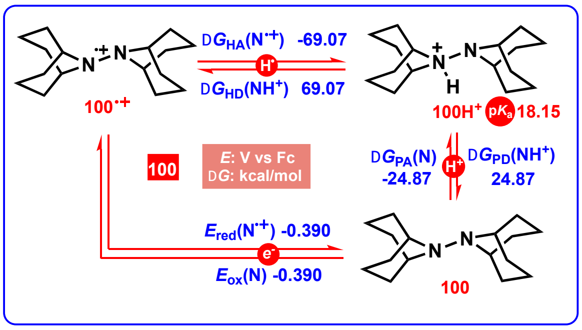

100
E: V vs Fc
DG: kcal/mol
DGHA(N•+) -69.07
DGHD(NH+) 69.07
100H+ pKa 18.15
DGPA(N) -24.87
DGPD(NH+) 24.87
Ered(N•+) -0.390
Eox(N) -0.390
100•+
100

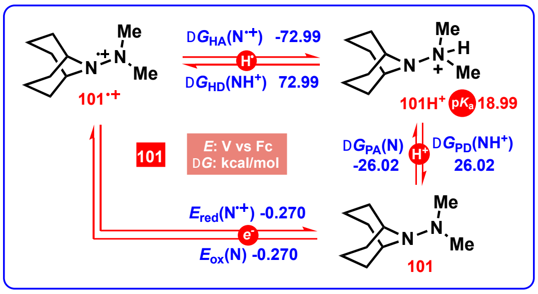
DGHA(N·+) -72.99
DGHD(NH+) 72.99
101·+
101H+ pKa 18.99
101
E: V vs Fc
DG: kcal/mol
DGPA(N) -26.02
DGPD(NH+) 26.02
Ered(N·+) -0.270
Eox(N) -0.270
101

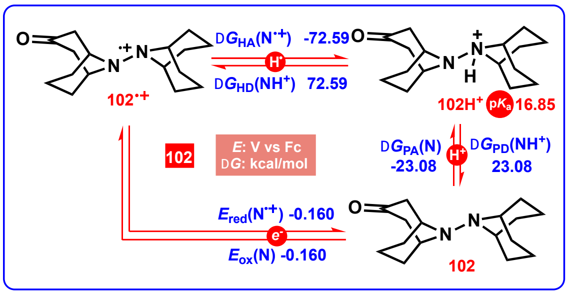
DGHA(N·+) -72.59
DGHD(NH+) 72.59
102·+
102H+ pKa 16.85
102
E: V vs Fc
DG: kcal/mol
DGPA(N) -23.08
DGPD(NH+) 23.08
Ered(N·+) -0.160
Eox(N) -0.160
102

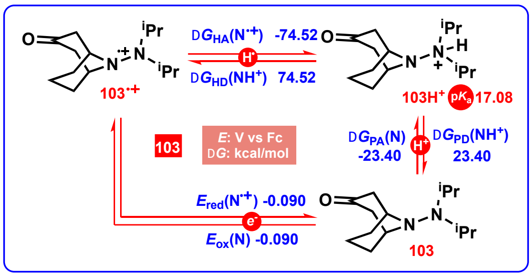
DGHA(N·+) -74.52
DGHD(NH+) 74.52
103·+
103H+ pKa 17.08
103
E: V vs Fc
DG: kcal/mol
DGPA(N) -23.40
DGPD(NH+) 23.40
Ered(N·+) -0.090
Eox(N) -0.090
103

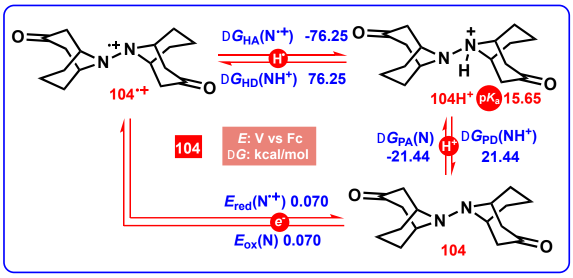
DGHA(N·+) -76.25
DGHD(NH+) 76.25
104·+
104H+ pKa 15.65
104
E: V vs Fc
DG: kcal/mol
DGPA(N) -21.44
DGPD(NH+) 21.44
Ered(N·+) 0.070
Eox(N) 0.070
104

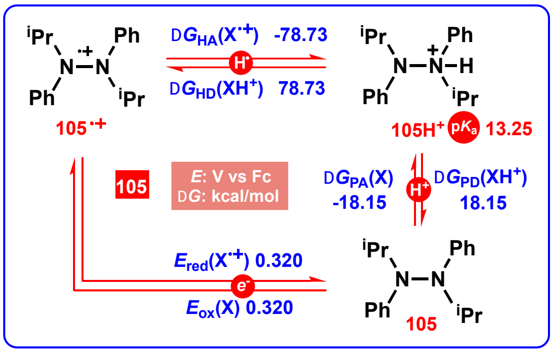
DGHA(X·+) -78.73
DGHD(XH+) 78.73
105·+
105H+ pKa 13.25
105
E: V vs Fc
DG: kcal/mol
DGPA(X) -18.15
DGPD(XH+) 18.15
Ered(X·+) 0.320
Eox(X) 0.320
105

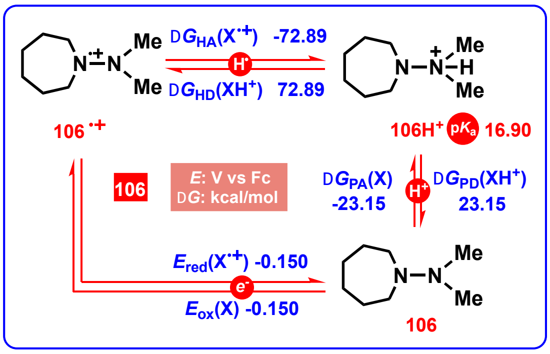
DGHA(X·+) -72.89
DGHD(XH+) 72.89
106·+
106H+ pKa 16.90
106
E: V vs Fc
DG: kcal/mol
DGPA(X) -23.15
DGPD(XH+) 23.15
Ered(X·+) -0.150
Eox(X) -0.150
106

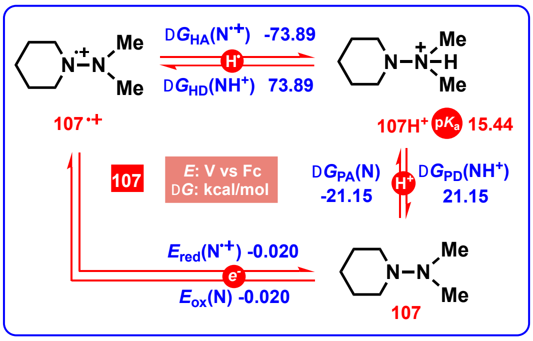
DGHA(N·+) -73.89
DGHD(NH+) 73.89
107·+
107H+ pKa 15.44
107
E: V vs Fc
DG: kcal/mol
DGPA(N) -21.15
DGPD(NH+) 21.15
Ered(N·+) -0.020
Eox(N) -0.020
107

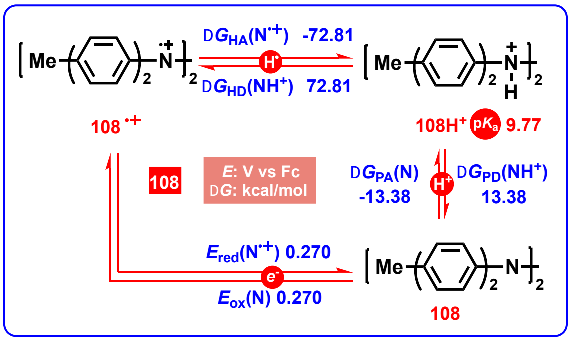
DGHA(N·+) -72.81
DGHD(NH+) 72.81
108·+
108H+ pKa 9.77
108
E: V vs Fc
DG: kcal/mol
DGPA(N) -13.38
DGPD(NH+) 13.38
Ered(N·+) 0.270
Eox(N) 0.270
108

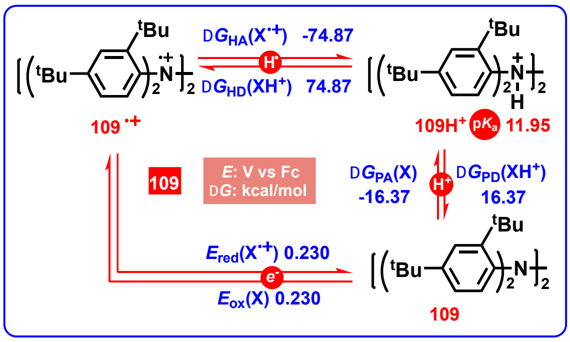
DGHA(X·+) -74.87
DGHD(XH+) 74.87
109·+
109H+ pKa 11.95
109
E: V vs Fc
DG: kcal/mol
DGPA(X) -16.37
DGPD(XH+) 16.37
Ered(X·+) 0.230
Eox(X) 0.230
109

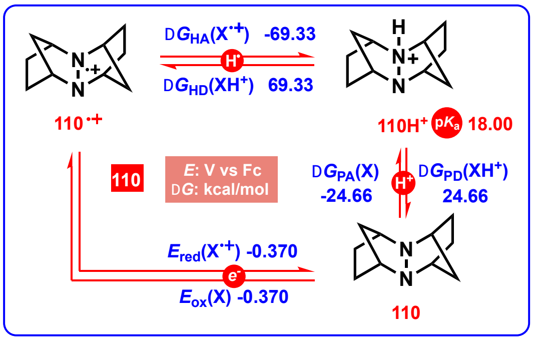
DGHA(X·+) -69.33
DGHD(XH+) 69.33
110·+
110H+ pKa 18.00
110
E: V vs Fc
DG: kcal/mol
DGPA(X) -24.66
DGPD(XH+) 24.66
Ered(X·+) -0.370
Eox(X) -0.370
110

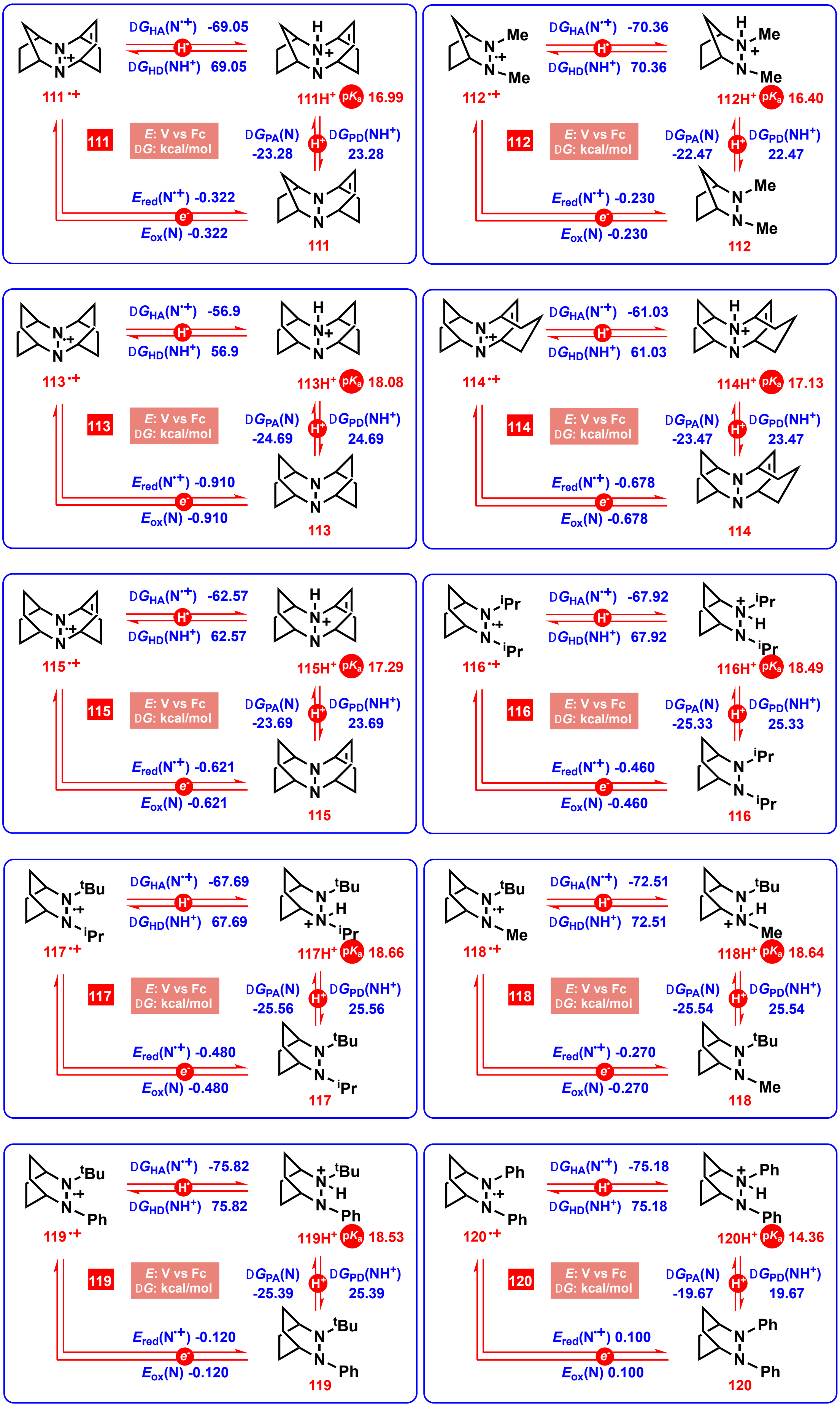

DGHA(N•+) -69.05
DGHD(NH+) 69.05
111•+
111H+ pKa 16.99
111
E: V vs Fc
DG: kcal/mol
DGPA(N) -23.28
DGPD(NH+) 23.28
Ered(N•+) -0.322
Eox(N) -0.322
111
DGHA(N•+) -70.36
DGHD(NH+) 70.36
Me
112•+
112H+ pKa 16.40
112
E: V vs Fc
DG: kcal/mol
DGPA(N) -22.47
DGPD(NH+) 22.47
Ered(N•+) -0.230
Eox(N) -0.230
112
DGHA(N•+) -56.9
DGHD(NH+) 56.9
113•+
113H+ pKa 18.08
113
E: V vs Fc
DG: kcal/mol
DGPA(N) -24.69
DGPD(NH+) 24.69
Ered(N•+) -0.910
Eox(N) -0.910
113
DGHA(N•+) -61.03
DGHD(NH+) 61.03
114•+
114H+ pKa 17.13
114
E: V vs Fc
DG: kcal/mol
DGPA(N) -23.47
DGPD(NH+) 23.47
Ered(N•+) -0.678
Eox(N) -0.678
114
DGHA(N•+) -62.57
DGHD(NH+) 62.57
115•+
115H+ pKa 17.29
115
E: V vs Fc
DG: kcal/mol
DGPA(N) -23.69
DGPD(NH+) 23.69
Ered(N•+) -0.621
Eox(N) -0.621
115
DGHA(N•+) -67.92
DGHD(NH+) 67.92
iPr
116•+
116H+ pKa 18.49
116
E: V vs Fc
DG: kcal/mol
DGPA(N) -25.33
DGPD(NH+) 25.33
Ered(N•+) -0.460
Eox(N) -0.460
116
DGHA(N•+) -67.69
DGHD(NH+) 67.69
tBu
iPr
117•+
117H+ pKa 18.66
117
E: V vs Fc
DG: kcal/mol
DGPA(N) -25.56
DGPD(NH+) 25.56
Ered(N•+) -0.480
Eox(N) -0.480
117
DGHA(N•+) -72.51
DGHD(NH+) 72.51
tBu
Me
118•+
118H+ pKa 18.64
118
E: V vs Fc
DG: kcal/mol
DGPA(N) -25.54
DGPD(NH+) 25.54
Ered(N•+) -0.270
Eox(N) -0.270
118
DGHA(N•+) -75.82
DGHD(NH+) 75.82
tBu
Ph
119•+
119H+ pKa 18.53
119
E: V vs Fc
DG: kcal/mol
DGPA(N) -25.39
DGPD(NH+) 25.39
Ered(N•+) -0.120
Eox(N) -0.120
119
DGHA(N•+) -75.18
DGHD(NH+) 75.18
Ph
120•+
120H+ pKa 14.36
120
E: V vs Fc
DG: kcal/mol
DGPA(N) -19.67
DGPD(NH+) 19.67
Ered(N•+) 0.100
Eox(N) 0.100
120
